# Impact of Additives on Drug Particles during Liquid Antisolvent Crystallization and Subsequent Freeze-Drying

**DOI:** 10.1021/acs.oprd.3c00204

**Published:** 2023-10-06

**Authors:** Peuli Ghosh, Ake Rasmuson, Sarah P. Hudson

**Affiliations:** † SSPC, the Science Foundation Ireland Research Centre for Pharmaceuticals, Department of Chemical Sciences, Bernal Institute, University of Limerick, Limerick V94 T9PX, Ireland; ‡ Department of Chemical Engineering, KTH Royal Institute of Technology, Stockholm SE-100 44, Sweden

**Keywords:** antisolvent crystallization, additive, freeze-drying, morphology, dissolution rate

## Abstract

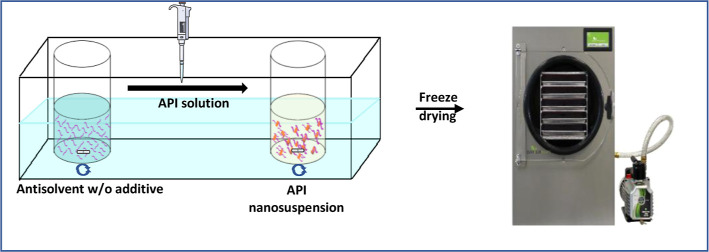

The impact of single or combinations of additives on the generation of nanosuspensions of two poorly water-soluble active pharmaceutical ingredients (APIs), fenofibrate (FF) and dalcetrapib (DCP), and their isolation to the dry state via antisolvent (AS) crystallization followed by freeze-drying was explored in this work. Combinations of polymeric and surfactant additives such as poly(vinyl alcohol) or hydroxypropyl methyl cellulose and sodium docusate were required to stabilize nanoparticles (∼200–300 nm) of both APIs in suspension before isolation to dryness. For both FF and DCP, multiple additives generated the narrowest, most-stable particle size distribution, with the smallest particles in suspension, compared with using a single additive. An industrially recognized freeze-drying process was used for the isolation of these nanoparticles to dryness. When processed by the liquid AS crystallization followed by freeze-drying in the presence of multiple additives, a purer monomorphic powder for FF resulted than when processed in the absence of any additive or in the presence of a single additive. It was noted that all nanoparticles freeze-dried in the presence of additives had a flat, flaky habit resulting in large surface areas. Agglomeration occurred during freeze-drying, resulting in micron-size particles. However, after freeze-drying, powders produced with single or multiple additives showed similar dissolution profiles, irrespective of aging time before drying, thus attenuating the advantage of multiple additives in terms of size observed before the freeze-drying process.

## 1 Introduction

It is estimated that almost 90% of new active pharmaceutical ingredients (APIs) have low water solubility, and although numerous existing preformulation and formulation strategies have been reported to increase water solubility, nearly 40% of these APIs fail to reach commercialization solely for this reason.^1−3^ An API must dissolve in the body, as well as permeate through biological barriers, to enter systemic circulation and achieve good bioavailability after administration. This can be particularly challenging when a medicine is taken orally due to the varying pH and composition of the gastrointestinal fluids and patient-to-patient variability. To date, strategies that have been developed to address the problem of poor water solubility include crystal modification,^4,5^ solid dispersions,^6^ polymeric micelle formation,^7^ lipid-based drug delivery,^8^ or particle size reduction.^1^ Decreasing the size of poorly water-soluble API particles leads to an increase in the surface area to volume ratio, which results in faster dissolution rates in aqueous media.^9^ Small particle sizes can be achieved by size reduction or controlled crystallization methods. The size reduction approach involves fragmentation of large particles to smaller counterparts by providing mechanical energy through processes such as wet-milling^3,10^ or high-pressure homogenization.^11,12^ At present, the size reduction approach is by far the most favored method to produce micro-/nanoparticles commercially.^13^ APIs such as sirolimus, aprepitant, fenofibrate (FF), and megestrole acetate with trade names Rapamune, Emend, Tricor, and Megace ES, respectively, are commercially available as API nanoparticle formulations produced by wet ball milling.^14^ Conversely controlled precipitation methods, such as antisolvent (AS) crystallization, while reported regularly in the literature, have not been adopted commercially by pharmaceutical manufacturing industries. AS crystallization is a fast, cost- and energy-efficient crystallization process that can produce API nanoparticles. It involves the generation of a solution that is highly supersaturated due to the low solubility of the API in the solvent and AS mixture. It has the potential to be scaled up industrially using continuous processes.^15^ Nanosuspensions can be prepared via continuous AS crystallization with the help of different techniques to mix the solvent and the AS solution, for example, by ultrasonically driven mixing devices,^16^ ultrasound-assisted microfluidics,^17^ droplet-based microreactors,^18^ or spinning disc reactors.^19^

Generally, soluble additives such as surfactants or polymers are used during an AS crystallization to stabilize the particle size distribution (PSD) of the resulting suspension^20^ or to control polymorphic transformations.^21^ These two processes can be linked at times with a polymorphic transformation resulting in particle growth.^22^ When the goal is to generate and maintain a small particle size, the additive is required to both promote nucleation or at least not hamper it and to inhibit crystal growth or transformation, depending on the target crystal form of the particles. It has been observed in several nucleation and crystal growth studies, over a limited supersaturation range, that single additives often cannot achieve both the promotion of nucleation and inhibition of crystal growth/transformation.^23−25^ Bodnar et al.^21^ studied the effect of additives on the nucleation of mefenamic acid and developed a novel strategy of nanoparticle production and stabilization by separately targeting nucleation and crystal growth steps with stabilizers. Mefenamic acid AS crystallization in the presence of sodium docusate (DOSS) enhanced the nucleation rate and the addition of the polymeric additive poly(vinyl alcohol) (PVA) or, hydroxypropylmethylcellulose (HPMC), five seconds after nucleation prevented further particle growth, helping to stabilize the particle size of the generated nanosuspension.

Nanosuspensions have some drawbacks which include long-term physical and chemical instability^26^ resulting in short shelf life periods.^26−29^ Also, nanosuspensions produced by liquid AS crystallization tend to have low API concentrations and, thus, may require large dose volumes to achieve an effective dose. Thus, separating nanoparticles from suspension into a dry powder form is often desired but is not without its challenges due to slow filtration rates and particle growth or agglomeration during separation. If the API nanoparticles can be dried without aggregation or agglomeration or loss of wettable surface area, then the dried powder will maintain its fast dissolution properties. Common nanosuspension isolation techniques include freeze-drying,^30^ spray drying,^31^ carrier particle-mediated isolation,^32^ spray-freeze-drying,^33^ or deposition as a coating.^34^ Spray drying is a process during which the suspension is atomized into fine droplets by a nozzle and evaporated using a hot drying gas.^35,36^ For spray drying processes, fast drying rates, that require higher temperatures, are desired as slow drying rates may produce stickier particles which lead to wall deposition and low yield.^37^ Spray drying can also be used as a separation technique to produce dried API nanoparticles in a continuous process.^13,38,39^

Freeze-drying, also known as lyophilization or cryodesiccation, can also be employed as an isolation technique to separate nano-/microparticles from suspension. During the freeze-drying process, nanosuspensions are typically frozen at normal pressure and then gradually heated under vacuum to produce a dried material.^40^ While freezing can prevent diffusion and crystal growth, it can cause nanoparticle aggregation and fusion due to the capillary stresses caused during the process.^36^ Thus, preventing agglomeration and achieving redispersibility are two of the main challenges faced during isolation to dryness using this method. The problem can be resolved by incorporating a good redispersant into the nanosuspension prior to freeze-drying that can rapidly dissolve upon contact with water or gastric fluid and redisperse the agglomerated nanoparticles.^40,41^ Normally sugars such as glucose, mannitol, or sucrose and polymers such as polyvinylpyrrolidone (PVP), Poloxamer 188 (Pluronic F68), or HPMC are used as redispersants, and they can also act as cryoprotectants during the freezing process.^30,40,42^ Kim et al. used HPMC as a stabilizer to produce a *trans*-resveratrol nanosuspension via a temperature-controlled AS crystallization and employed freeze-drying to produce dried nanoparticles.^43^ This study showed that any extra usage of cryoprotectants was unnecessary to suppress agglomeration, indicating the ability of HPMC to stabilize the nanoparticles during nucleation, growth, and drying. Although a slight increment in particle size after freeze-drying was observed, the produced dried particles stayed within the nanosize range with HPMC only.^43^ Tierney et al. used an AS crystallization process in the presence of the soluble polymeric additive PVA to generate a stable nanosuspension of FF for up to 11 min and isolated the FF particles to dryness via freeze-drying.^44^ During the freeze-drying, micron-size FF particles preserved their particle size but submicron-size particles agglomerated to larger sizes.^44^ Although several studies to produce FF nanosuspensions^44,45^ via AS crystallization have been reported, there is still little understanding of the effect of combinations of additives on particle size stabilization and subsequent freeze-drying processes.

The carrier particle-mediated filtration process is a relatively new, efficient, and cost-effective way to isolate nanoparticles from suspension in which insoluble carrier particles are added to the AS and nanoparticles are adsorbed to its surface resulting in easy separation through filtration. Tierney et al. and Bodnar et al. used montmorillonite clay (MMT) as a carrier particle to isolate fast-dissolving FF, mefenamic acid, and dalcetrapib (DCP) nanocomposite powders with up to 9.1, 4.8, and 20.9% API loading, respectively.^46,47^ Kumar et al. used a semicontinuous method to produce gram-scale valsartan nanocomposite suspensions in a 60 mL mixing chamber using the previously used MMT clay carrier particles and isolated the powders to dryness using a simple in-line Buchner funnel setup.^48^ This carrier particle-mediated isolation approach has not yet been used commercially. In addition, spray-drying, while becoming more accepted to the industry, frequently results in low yields.^37^ This leaves freeze-drying as one of the more industrially accepted methods to isolate nanoparticles to dryness.

**Figure 1:**
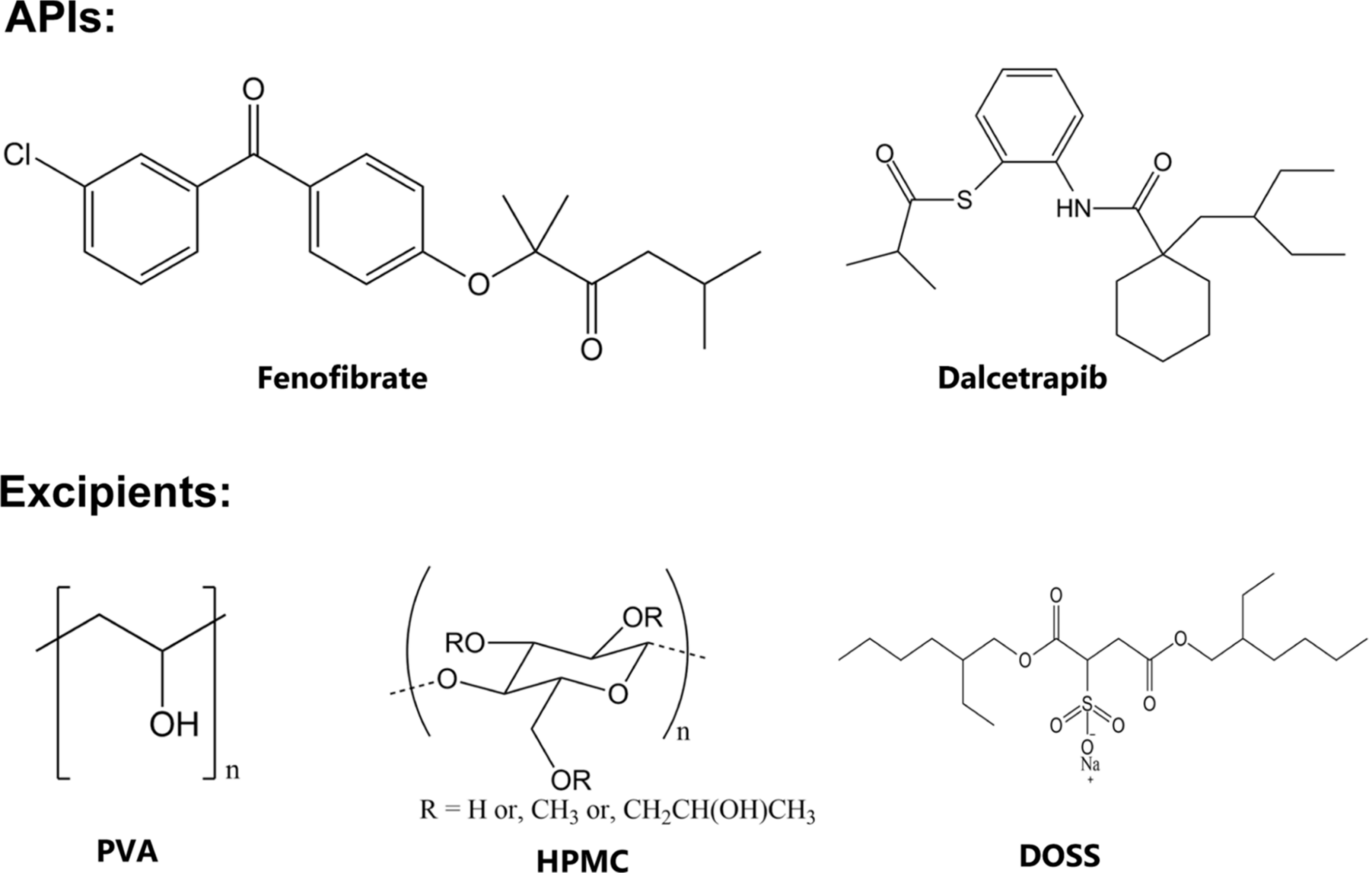
Chemical structure of APIs and excipients used for the production of a rapidly dissolving dry API powders.

There are limited data on the role of stabilizer combinations used to generate stable dry API nanoparticles. The studies described above indicate the potential of timely addition of additives to separately target and control the nucleation, growth, and drying of API suspensions in an effort to produce stable, dry API nanoparticles. However, additional studies are needed to confirm the feasibility of this approach. This work focuses on AS crystallization of two poorly water-soluble APIs, FF and DCP, as shown in Figure 1, with the aid of combinations of additives to generate stable nanosuspensions with high dissolution rates. Industrially recognized freeze-drying procedures, which produce a higher yield than spray drying,^37^ are used to isolate the generated suspensions to dry powder and dissolution properties of these dried samples are studied to determine if the additives that controlled the particle size during crystallization can continue to stabilize the nanoparticles during the subsequent freeze-drying process. The main objective of the work is to generate dry API powders with fast dissolution kinetics using existing approved manufacturing processes.

## 2 Experimental Section

### 2.1 Materials

FF (99.7% purity) and DCP (99.4% purity) were generously gifted by Abbott Laboratories and Roche, respectively. Methanol (MeOH, 99.5% purity) was purchased from Merck Millipore. Additives: PVA (9–10 kDa), HPMC (∼26 kDa), PVP K30 (40 kDa), sodium dodecyl sulfate (SDS), DOSS, and Polysorbate 80 (Tween 80) were all purchased from Sigma-Aldrich. Deionized water was used for aqueous solutions (18 MΩ; Elga, Purelab Ultra). All chemicals were used as received without further purification.

### 2.2 Preparation of API Suspensions and Isolation to Dryness

An undersaturated FF solution (50 mg/mL) was prepared by adding 0.5 g of solute to a 20 mL glass vial with a polytetrafluoroethylene (PTFE) seal containing 10 mL of MeOH. The glass vial was placed in a water bath at 20 °C and stirred at 1200 rpm using a magnetic stir bar for 2 h. The resulting solution was then filtered using a 0.22 μm pore size PTFE filter to remove any insoluble impurities. FF solutions at 5 °C and DCP solutions at 25 and 5 °C were prepared using the same method at concentrations of 17, 60, and 21 mg/mL, respectively, and all solutions were undersaturated under these conditions.

AS stock solutions were prepared in a 100 mL glass bottle containing distilled water and additives at specified concentrations, as provided in Table 1. The stock solution was placed in a water bath at 25 °C and stirred at 1200 rpm for 2 h. A specific volume of this AS solution was transferred to a 20 mL glass vial containing a 13 mm magnetic stir bar and was placed in a water bath at the specified temperature according to Table 1 and left to stir at 1200 rpm for 1 h.

**Table 1:** Process Conditions for the AS Crystallization of FF and DCP in the Presence and Absence of Single and Multiple Soluble Additives

API	temperature	additive system	additive conc. In AS (w/v %)	AS/S ratio (v/v ratio)^a^	API/additive (w/w ratio)
FF	20 °C	no additive	NA	10:1	
			NA	47:3	
	5 °C	no additive	NA	47:3	
		PVA	0.1	47:3	1.1:1
		DOSS	0.05	47:3	2.2:1
		HPMC	0.1	47:3	1.1:1
		DOSS + PVA	0.05 and 0.1	47:3	2.2:1 (DOSS)
					1.1:1 (PVA)
		DOSS + HPMC	0.05 and 0.1	47:3	2.2:1 (DOSS)
					1.1:1 (PVA)
		DOSS 10 s later PVA	0.05 and 1	47:3	2.2:1 (DOSS)
					1:1 (PVA)
		DOSS 10 s later HPMC	0.05 and 1	47:3	2.2:1 (DOSS)
					1:1 (PVA)
DCP	25 °C	no additive	NA	47:3	
	5 °C	no additive	NA	47:3	
		PVA	0.1	47:3	6.7:5
		HPMC	0.1	47:3	6.7:5
		DOSS	0.05	47:3	6.7:2.5
		DOSS + HPMC	0.05 and 0.1	47:3	6.7:5 (DOSS)
					6.7:2.5 (HPMC)
		DOSS + PVA	0.05 and 0.1	47:3	6.7:5 (DOSS)
					6.7:2.5 (PVA)
		DOSS 10 s later HPMC	0.05 and 1	47:3	6.7:2.5 (DOSS)
					6.3:5 (HPMC)
		DOSS 10 s later PVA	0.05 and 1	47:3	6.7:2.5 (DOSS)
					6.3:5 (PVA)

a Ratio of AS/S at the point of nucleation.

Nanosuspensions were prepared by using a previously reported liquid AS crystallization method.^44,47,49^ An aliquot of the API solution (0.3 mL) was taken by an Eppendorf pipet and quickly introduced to the AS solution (4.7 mL) under the surface at constant temperature with stirring at 1200 rpm throughout the crystallization process. A series of AS crystallizations of FF and DCP were conducted in the presence of different single additives PVA, DOSS, SDS, HPMC, PVP k-30, and Tween 80 at a range of concentrations (0.1–0.2 w/v %).

Subsequently, AS crystallizations of FF and DCP were conducted in the presence of combinations of DOSS with HPMC or PVA in the AS. Additives were either (i) both present during the nucleation or (ii) added in a stepwise fashion where a single additive was present during nucleation, followed by addition of the second additive (0.5 mL) 10 s after introduction of the API solution (0.3 mL) to the AS containing the first additive (4.7 mL), as indicated in Table 1. Process conditions including the additive concentration, AS/S ratio, and temperature were varied, as listed in Table 1.

Suspensions prepared at 5 °C were isolated and dried via freeze-drying (Severn LS40). The suspensions (5 mL) at different aging times such as 10, 20, and 30 min were flash frozen in liquid nitrogen, followed by freeze-drying under vacuum (<27 Pa) for 24 h with an initial freezing temperature at −25 °C for 3 h and then slowly increased to a final temperature of 20 °C over 24 h.

### 2.3 Particle Size and Zeta Potential Measurements of API Suspensions

The PSDs of suspensions were measured by laser diffraction on a Malvern Mastersizer 3000 system, with water as the dispersion medium. In each experiment, after a certain time called “aging time” (30 s, 10, 20, 30, and 60 min) after the addition of the API solution to the AS, ∼1–2 mL of the suspension was extracted and added to the sample addition chamber until an obscuration value of ∼4–7% was reached. The sample was dispersed in water for 30 s with a stirring rate of 2000 rpm for each measurement. Each sample was measured with three consecutive sub runs using a 10 s delay between runs. All sub runs generated similar results. Measurement time for each sub run was 40 s (20 s each for blue laser and red laser). The size distribution of FF and DCP suspensions was calculated with refractive indices of 1.55 and 1.543, respectively, and an absorption index of 0.01 for both. The volume-weighted mean diameter of *D*[4,3] was recorded for each measurement.

Particle size measurements for sizes below 400 nm were carried out on a Malvern Zetasizer Nano ZPS system. A refractive index of 1.543 and an absorption index of 0.01 were used to determine the size of the particles in the DCP nanosuspensions. Samples were equilibrated for 60 s at 5 °C before measurement. Each measurement consisted of 3 runs and 10 sub runs for 14 s each sub run. The average D_50_ diameter of three measurements was recorded for each sample.

PSDs for freeze-dried (FD) products resuspended in water were measured by laser diffraction on a Malvern Mastersizer 3000 system similar to the measurement performed on suspensions before drying. The suspension was prepared by adding 5 mg of dried nano-/microparticles to a 20 mL glass vial with PTFE seal containing 5 mL of water at 5 °C. The glass vial was placed in a water bath at 5 °C and stirred at 1200 rpm using a magnetic stir bar for 2 h. “As-received” and FD API particles produced without additives had very low wettability. Thus, a surfactant solution instead of deionized water was used to prepare a suspension of these samples for particle sizing. FF particles were resuspended in 0.4% w/v Tween-80, and 1.5 mg/mL SDS solution was used to prepare DCP suspensions. Each set of experiments was performed three times, and the average with standard deviation was reported.

The ζ potential was measured by using a Malvern Zetasizer Nano ZSP system. A folded capillary cell of cell-type DTS1070 was used to perform the measurements. Around 1 mL of the suspension after 1 min aging time was inserted into the cell, and the temperature was equilibrated for 120 s at 25 °C prior to the measurement. Each sample was measured three times with 20 consecutive sub runs, and the average with standard deviation was recorded.

### 2.4 Solid-State Characterization of Dried Particles

The crystalline form and degree of crystallinity of “as-received” APIs and the FD particles were determined by powder X-ray diffraction (PXRD). A Philips PANalytical X’pert diffractometer was used to record the diffraction pattern by using Ni-filtered Cu Kα radiation (λ = 1.5406 Å) in reflection mode at 40 kV and 40 mA. The diffraction patterns for all FF and DCP samples were collected, respectively, between 5–40° and 5–30° (2θ) with 0.026° 2θ/min step size and 60 s per step.

Thermal analysis was carried out using a Netzsch Polyma 214 differential scanning calorimeter in the range of 20–100 °C under a constant nitrogen environment (30 mL/min). Experiments were performed by placing approximately 3–5 mg of the powder sample into an aluminum pan and sealing it with an aluminum lid with a pinhole using a press. Samples were analyzed at a scan rate of 10 K/min, and the instrument was calibrated using indium and lead.

A Hitachi SU-70 high-resolution scanning electron microscope was used to analyze sample habit. A small amount of FD powder was put on the adhesive carbon tape and coated with a gold deposit using an EMITECH K55. All images were taken in field-free mode with a 10 kV voltage and a working distance of 15 mm. All SEM images were representative of the entire sample analyzed in each case.

### 2.5 Dissolution Rate Determination

The dissolution media (DM) used were a 0.1 M HCl solution containing 0.4% w/v Tween-80 for FF and a 0.1 M HCl solution containing 2 g/L NaCl and 2.5 g/L SDS for DCP, respectively. Dissolution testing for FF samples was carried out under sink conditions with a maximum concentration equivalent to 27.7 μg/mL in 100 mL of DM at a constant stirring rate of 300 rpm and a temperature of 42 °C. At specific time intervals, ∼2–3 mL aliquots of the DM were withdrawn using preheated plastic syringes to 50 °C and filtered through PTFE syringe filters with 0.22 μm pore size. The first 1.5 mL aliquot was discarded to avoid the absorption of FF on the PTFE membrane. The remaining volume was used to determine the API concentration in the DM via measuring the absorption in a Shimadzu UV-1800 UV–visible spectrophotometer at λ = 288 nm.

For DCP samples, in vitro dissolution testing was performed by adding enough sample to equate to 2.5 mg of DCP per 100 mL of DM (sink conditions) at 300 rpm constant stirring rate and 37 °C. The extent of API dissolution was measured using a similar process to that used for FF, with a UV–vis spectrometer at a wavelength, λ, of 244 nm, and solutions were filtered through a nylon syringe filter before analysis. Dissolution experiments were performed in triplicate, and the averages of the three concentrations obtained at each different time point from each experiment are reported, with standard deviations

## 3 Results

### 3.1 PSD of API Suspensions Prepared by Liquid AS Crystallization

#### 3.1.1 No Additives

**Figure 2:**
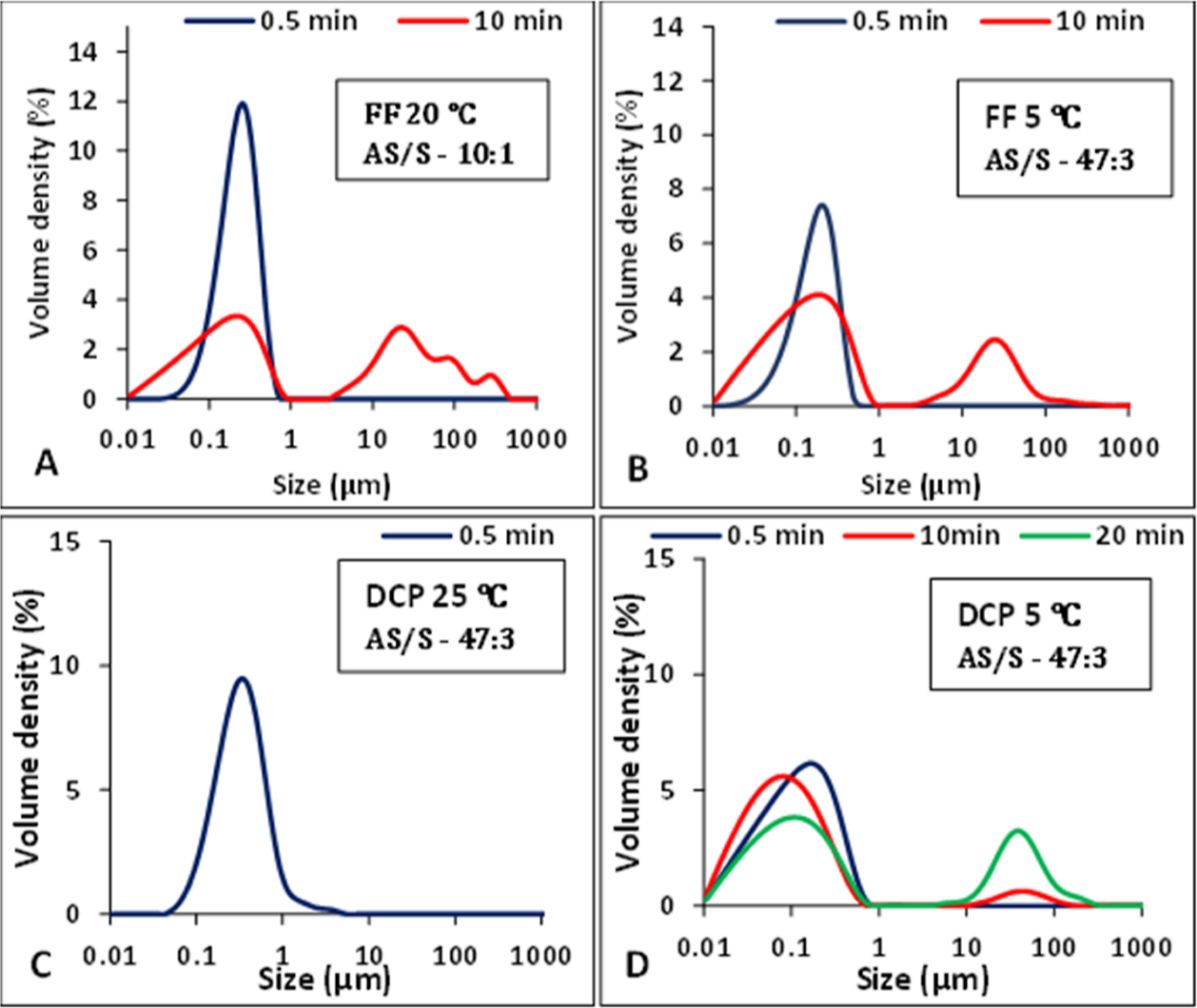
PSD at different times and temperatures in the absence of additives for (A) FF at 20 °C, (B) FF at 5 °C, (C) DCP at 25 °C, and (D) DCP at 5 °C.

Using the process conditions described in Table 1, after the introduction of the API solutions to aqueous AS without any additives, the solubility of both APIs swiftly decreased, causing a very high supersaturation level, which resulted in the formation of a milky white suspension. For FF at 20 °C after 30 s aging time, there was a unimodal PSD, as shown in Figure 2A, in the nanometer range. However, after 10 min aging time, there was a mix of nano- and micron-size particles and a bimodal PSD was observed, as shown in Figure 2A. After that, large particle aggregates settled out of suspension. The PSD for FF suspensions at 5 °C showed a similar trend. At 30 s and 10 min aging times, respectively, unimodal and bimodal PSDs were observed, as shown in Figure 2B.

The PSD for the DCP suspensions at both temperatures showed similar trends. At 25 °C, DCP suspensions had a unimodal PSD in the nanometer-size range (Figure 2C) after 30 s aging time, but due to the formation of larger agglomerates at longer aging times, particles started to settle out of suspension which prevented the measurement of the PSD. At 5 °C, a unimodal distribution at 30 s and bimodal distributions at 10 and 20 min aging time, respectively, were observed, as shown in Figure 2D. At 10 min aging time, the size of the second peak was small, but at 20 min aging time, it was larger due to an increase in micron-size particles in suspension. Subsequent crystallizations reported below were carried out at 5 °C.

#### 3.1.2 Single Additives

A series of AS crystallizations of FF and DCP were conducted in the presence of different single additives, PVA, DOSS, SDS, HPMC, PVP k-30, and Tween 80, at a range of concentrations (0.2–0.1 w/v %). DOSS, PVA, and HPMC showed the best stabilizing potential for these APIs (Supporting Information, Table S2).

Out of the different additives, the polymeric additive PVA and the surfactant additive, DOSS, generated the smallest-sized particles at the concentrations listed in Table 1 for both FF and DCP (Figure 4 and Supporting Information). When the polymeric additive HPMC was used, nanosized FF particles were detected for up to 10 min but thereafter grew in size, while micrometer-size DCP particles were present at 10 min (Supporting Information). For both APIs at 5 °C, in the presence of DOSS, a translucent suspension was formed initially for ∼20–30 s, whereas with PVA or HPMC, a milky white suspension was immediately observed.

**Figure 3:**
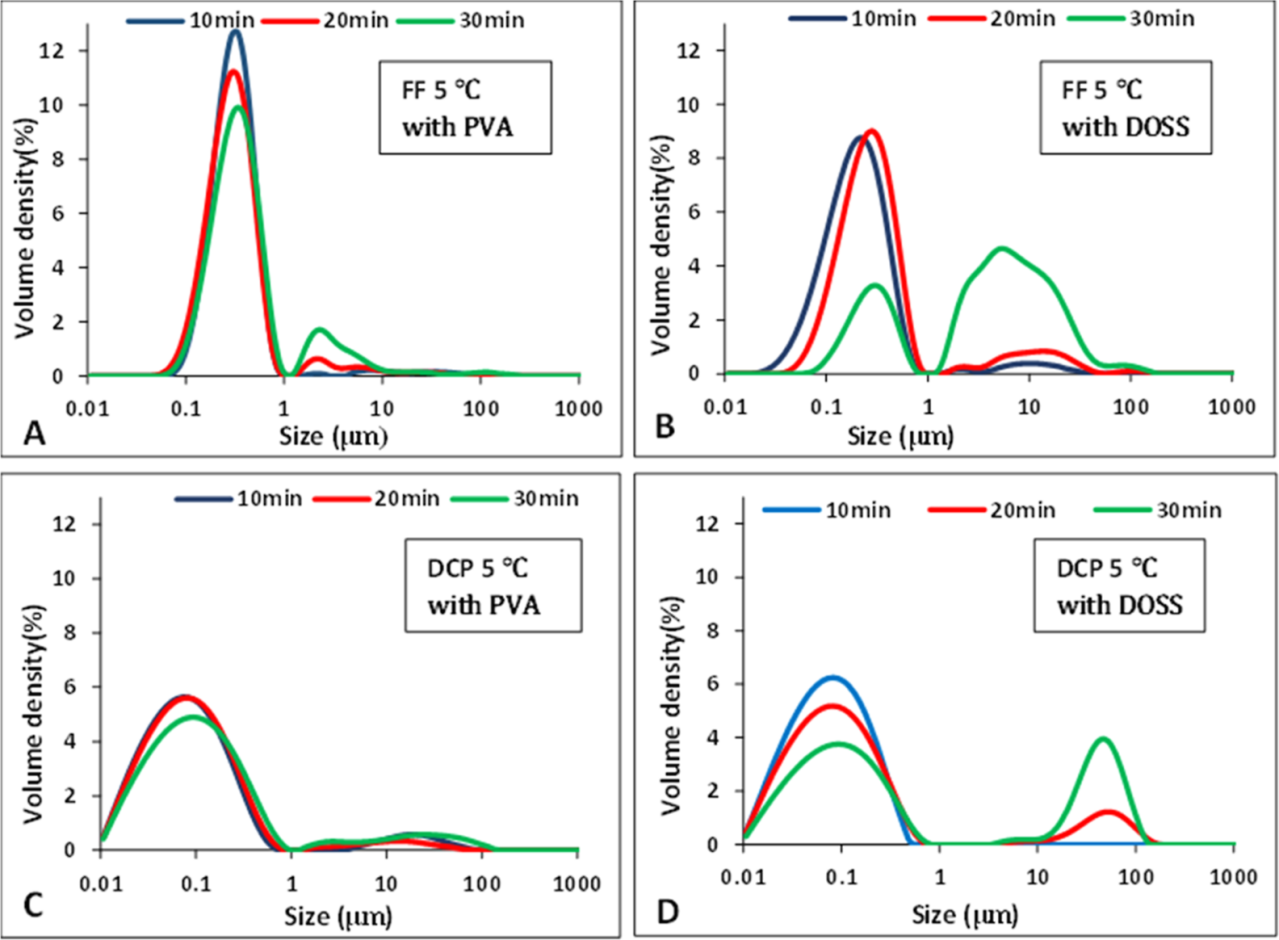
PSD of API suspensions over time with single additives at 5 °C for (A) FF with PVA, (B) FF with DOSS, (C) DCP with PVA, and (D) DCP with DOSS.

**Figure 4:**
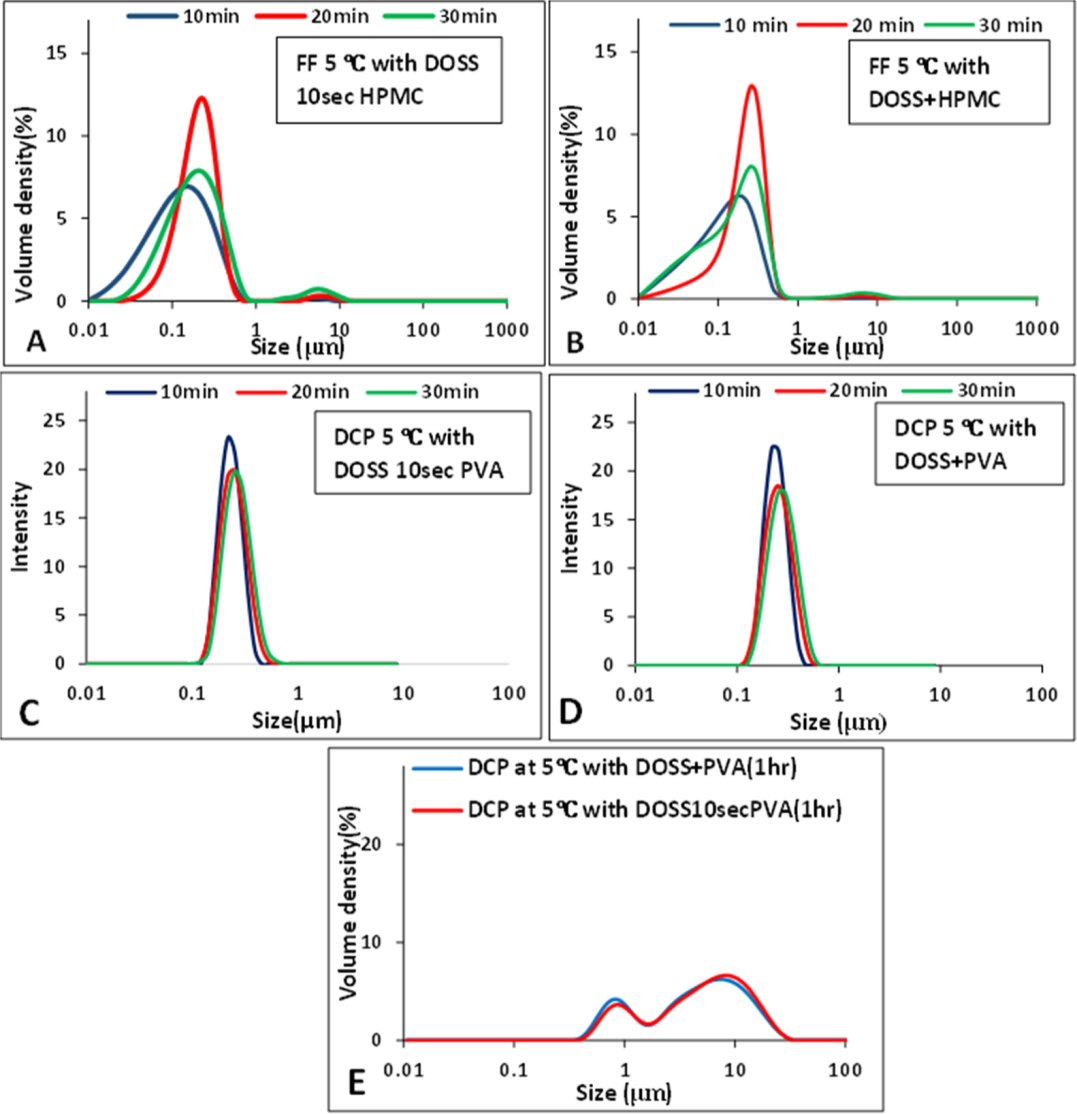
PSD of API suspensions with multiple additives at 5 °C over time. (A) FF with DOSS 10 s HPMC, (B) FF with DOSS + HPMC, (C) DCP with DOSS 10 s PVA, (D) DCP with DOSS + PVA measured by Zetasizer, and (E) DCP suspensions at 1 h aging time in the presence of multiple additives DOSS + PVA.

For FF suspensions in the presence of PVA at 10 min aging time, a unimodal distribution in the nanometer range was observed (Figure 3A). However, after 20 min aging time, the particles in suspension convert into a bimodal distribution containing a mix of nano- and micron-size particles with the latter peak being smaller. For FF suspensions in the presence of DOSS from 10 min, a bimodal distribution was observed (Figure 3B). The size of the second peak started to increase with time due to an increase of micrometer-size particles in suspension. Thus, PVA can stabilize FF suspension particle size better than DOSS over time.

Similar PSD trends were also observed for the DCP suspensions. In the presence of PVA, the PSD was bimodal after 10 min aging time, but the second peak remained small over time (Figure 3C). In the presence of DOSS at 10 min, a nanometer-range unimodal PSD was observed which transformed to a bimodal distribution after 20 min of aging time, and the size of the second peak in the micrometer range increased with time (Figure 3D). Thus, the polymeric additive, PVA, was better able to prevent the growth of the mean particle size over time than the surfactant additive DOSS for both APIs studied.

#### 3.1.3 Multiple Additives

FF suspensions generated at 5 °C in the presence of multiple additives DOSS and HPMC, as shown in Figure 4, had PSDs in a lower particle size range compared to when they were generated with multiple additives DOSS and PVA (Supporting Information). Adding the additives simultaneously or in a stepwise fashion did not have a large impact on the PSD. A unimodal PSD was observed at 10 min aging time which with time converted into a bimodal distribution. However, the size of the second peak was small at both 20 and 30 min aging times resulting in *D*[90] values in the nanometer range only. The presence of smaller particles at longer time frames can be attributed to attrition in the process, which needs further investigation. When this result is compared with the single additive PVA (Figure 3A), although the mean particle size of the suspension with PVA alone remains in the nanometer range, the volume fraction of the second peak was larger compared to that of multiple additives DOSS and HPMC (Figure 4A,B). This resulted in *D*[90] values for suspensions with PVA alone in the micrometer range (Supporting Information). The PSD of an FF suspension with PVA alone showed a smaller volume fraction second mode compared with multiple additives DOSS and PVA (Supporting Information) added in either manner at a 30 min aging time. Thus, the combination of DOSS and HPMC demonstrated the best stabilization for FF nanosuspensions for up to 30 min aging time.

**Figure 5:**
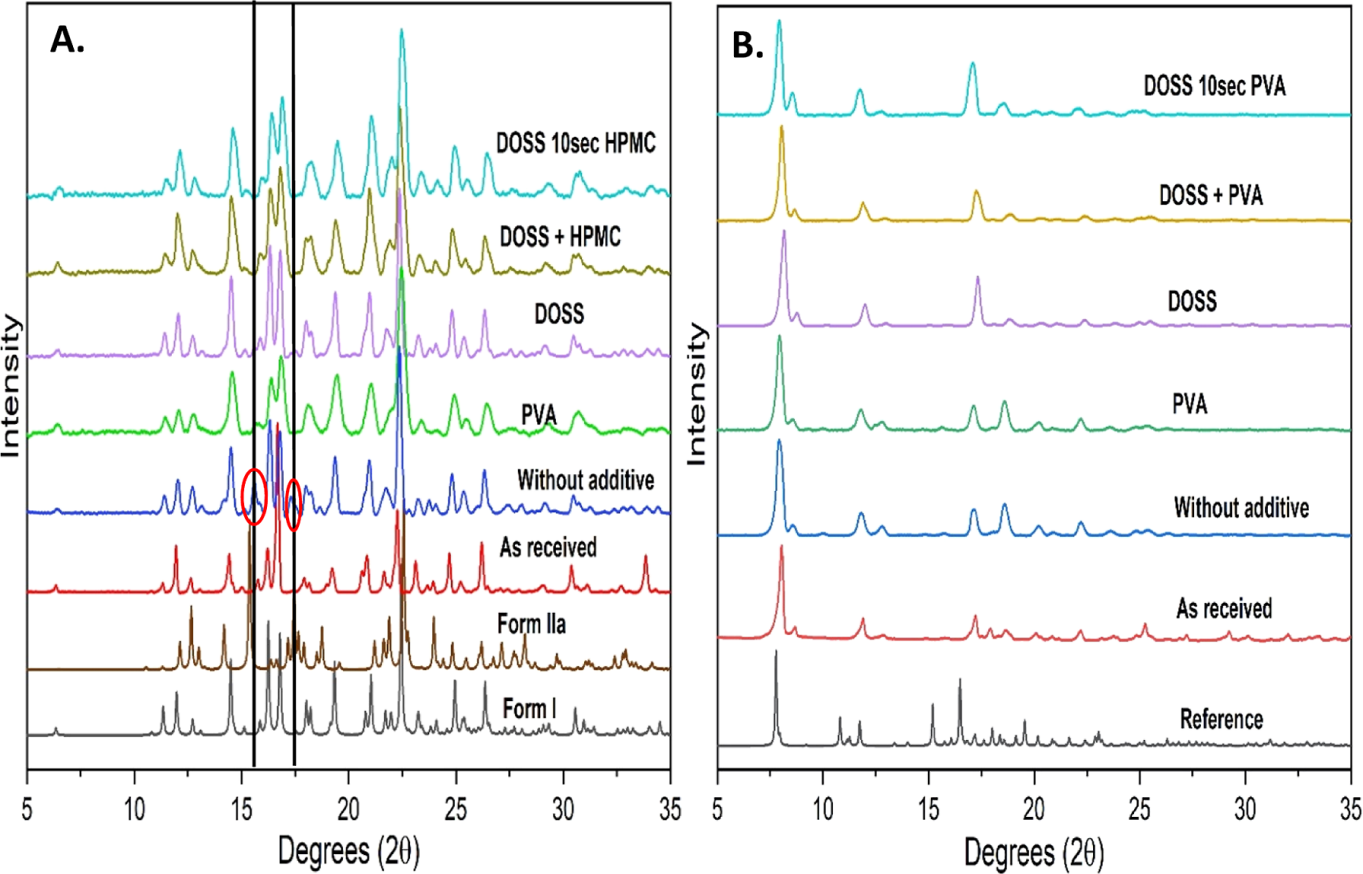
PXRD analysis of FD APIs at 10 min aging time precipitated in the presence or absence of additives at 5 °C (A) for FF with reference to form I (CCDC number 214632^50^) and form IIa (CCDC number 1822341^51^); peaks of form IIa showed by red circle; (B) for DCP with reference to form A (CCDC number 895592^52^).

Conversely, DCP suspensions generated at 5 °C in the presence of additives DOSS and PVA had a PSD in a lower nanometer range than DOSS and HPMC (Figure 4C,D and Supporting Information). Adding the additives simultaneously or in a stepwise fashion did not have a large impact on the PSD when the combination of PVA and DOSS was used with DCP, as shown in Figure 4C,D. A unimodal distribution in the presence of multiple additives DOSS + PVA and stepwise addition of DOSS 10 s PVA over a time of 30 min in nanometer range was observed (Figure 4C,D). At 1 h aging time, the suspension had a bimodal distribution for both multiple additives DOSS + PVA and stepwise addition of DOSS 10 s PVA with *D*[50] and *D*[90] values in the micrometer range (Figure 4E and Supporting Information). When comparing the PSDs of DCP nanoparticles in the presence of a single additive with multiple additives, it is clear that multiple additives can stabilize the particle size far better than single additives, but even multiple additives can only keep the particles in the nanometer range for up to 30 min.

Thus, it is evident that the right combination of multiple additives can better stabilize FF and DCP suspensions at 5 °C than any of the single additives tested. The mode of addition of multiple additives, that is simultaneously before nucleation or adding the polymeric additive in a stepwise fashion after nucleation, did not reduce the particle size or better stabilize it for the optimal combinations of multiple additives.

#### 3.1.4 Surface Charge

The zeta potential (ζ) for FF nanosuspensions generated without additives at 1 min aging time was −35 ± 2 mV, whereas in the presence of DOSS at 1 min aging time, it increased in magnitude to −44 ± 3 mV. With a nonionic polymeric additive (PVA or HPMC) or a mixture of surfactant and polymeric additives, it decreased to −4 ± 1 mV. Similarly, for DCP, the ζ potential of particles produced in the presence of DOSS increased from −39 ± 2 mV (without additive) to −59 ± 3 mV. Also, in the presence of multiple additives, it was lowest, −19 ± 2 mV. The increased zeta potential in the presence of the ionic additive DOSS provided electrostatic stabilization to the particles. This electrostatic stabilization would be lost or decreased in the presence of polymeric additives as the surface charge decreased toward 0 for FF and DCP, respectively. The polymeric additives, however, can provide steric stabilization, thus preventing agglomeration.

### 3.2 Solid-State Analysis of FD APIs

#### 3.2.1 Polymorphic Form

The solid state of the as-received and FD FF and DCP powders was characterized by PXRD, differential scanning calorimetry (DSC), and scanning electron microscopy (SEM) analysis. PXRD patterns of unprocessed (“as-received”) FF showed the characteristic high-intense diffraction peaks of the stable polymorphic form I confirmed with data from the Cambridge Structural Database (CCDC number 214632^50^), as shown in Figure 5A. The FD FF precipitated in the absence of additives showed peaks from both polymorphic form I and form II (CCDC number 1822341^51^). All FF samples that were FD in the presence of additives showed characteristic peaks of only stable form I. PXRD patterns of “as-received” DCP along with FD DCP in the presence or absence of additives showed characteristic peaks for stable polymorph form A (CCDC number 895592^52^). Although all peaks including those of “as-received” DCP were somewhat shifted toward higher angle compared to the peaks in reference-simulated PXRD from Cambridge structural database calculated at 100 K, all FD DCP peaks precipitated in the absence or presence of additives were in same position as the “as-received” DCP peaks, as shown in Figure 5B. DSC analysis of the pure additives (PVA, HPMC, and DOSS) shows that for PVA and DOSS, there were no overlapping thermal events with the melting of the APIs (Supporting Information). For HPMC, there is a thermal event overlapping with the melting of the APIs, but it gives a broad signal in the temperature range of 40–120 °C, differing from the sharp melting peaks of the APIs. The “as-received” FF thermogram showed an endothermic peak with the onset point at 80.8 ± 2.6 °C which corresponds to the melting point of the stable polymorphic form I.^53^

**Figure 6:**
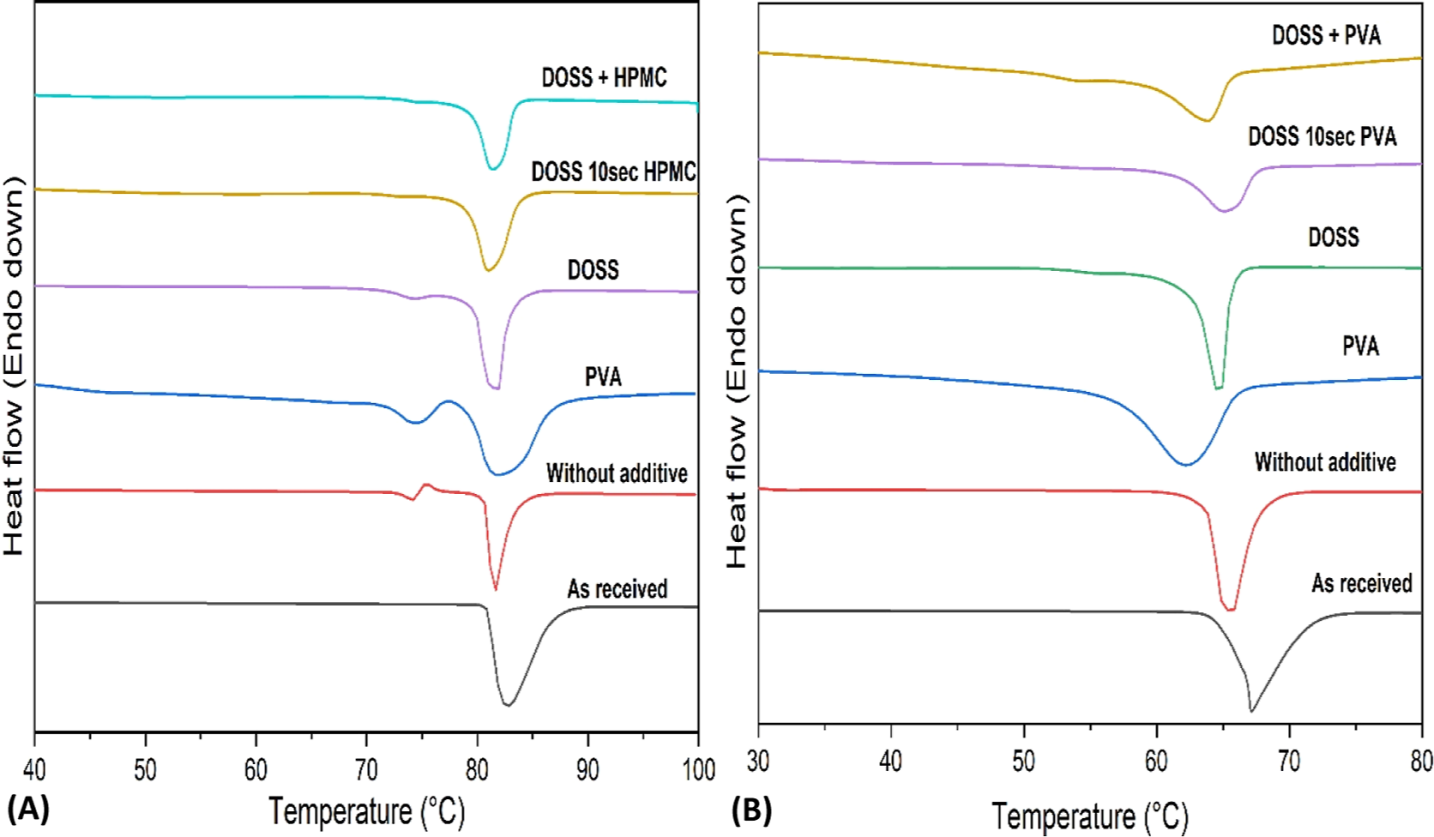
DSC analysis of FD APIs at 10 min aging time precipitated in the presence or absence of additives at 5 °C: (A) FF and (B) DCP.

The FD FF sample precipitated without additives showed endothermic peaks with onsets at 72.8 ± 3.7 and 80.2 ± 1.8 °C, as shown in Figure 6A. The first peak at 72.8 ± 3.7 °C corresponds to polymorphic form II,^54^ whereas the second peak at 80.2 ± 1.8 °C corresponds to form I. Also, FD FF samples precipitated with single additives, for example, DOSS or PVA, showed two endothermic peaks at 71.9 ± 1.4 and 78.9 ± 2.6, 72.4 ± 2.3 and 77.6 ± 3.1 °C, respectively, indicating the presence of both polymorphic forms (Figure 6A). FD FF in the presence of multiple additives DOSS + HPMC and DOSS 10 s HPMC had only a single endothermic peak at onset 78.5 ± 1.6 and 77.9 ± 2.5 °C corresponding to the stable polymorphic form I (Figure 6A).

Combining the results from both PXRD and DSC, it is concluded that FD FF samples precipitated in the absence of additives or with single additives containing both polymorphic form I and form II, whereas FD FF prepared in multiple additives contains only stable form I (Figures 5A and 6A). Thus, it is hypothesized that the presence of multiple additives enhanced the formation of stable polymorph form I during the AS crystallization process and/or quickened the transformation of the metastable form to the stable form during the AS crystallization and/or drying processes.

DSC thermograms of all FD DCP particles showed a single endothermic peak, as shown in Figure 6B. The onset melting temperature of pure, “as-received” DCP was 63.7 ± 4.9 °C at a heating rate of 20 K/min. For FD DCP prepared in the absence of additives, the onset melting temperature was 63.3 ± 3.2 °C. FD DCP precipitated in the presence of single additives PVA or DOSS had onset temperatures at 57.1 ± 4.6 and 61.9 ± 3.2 °C, respectively. FD DCP precipitated in the presence of multiple additives, DOSS + PVA or DOSS 10 s PVA, showed endothermic peak temperature onsets at 59.9 ± 4.5 and 61.4 ± 4.7 °C, respectively. Despite multiple analyses, the standard deviation observed in the melting temperatures for all DCP samples remained high, likely due to the slow heating rate used (10 K/min), but the melting temperatures measured were comparable to literature values and were not significantly different from each other.^46^

#### 3.2.2 PSD of FD APIs

**Figure 7:**
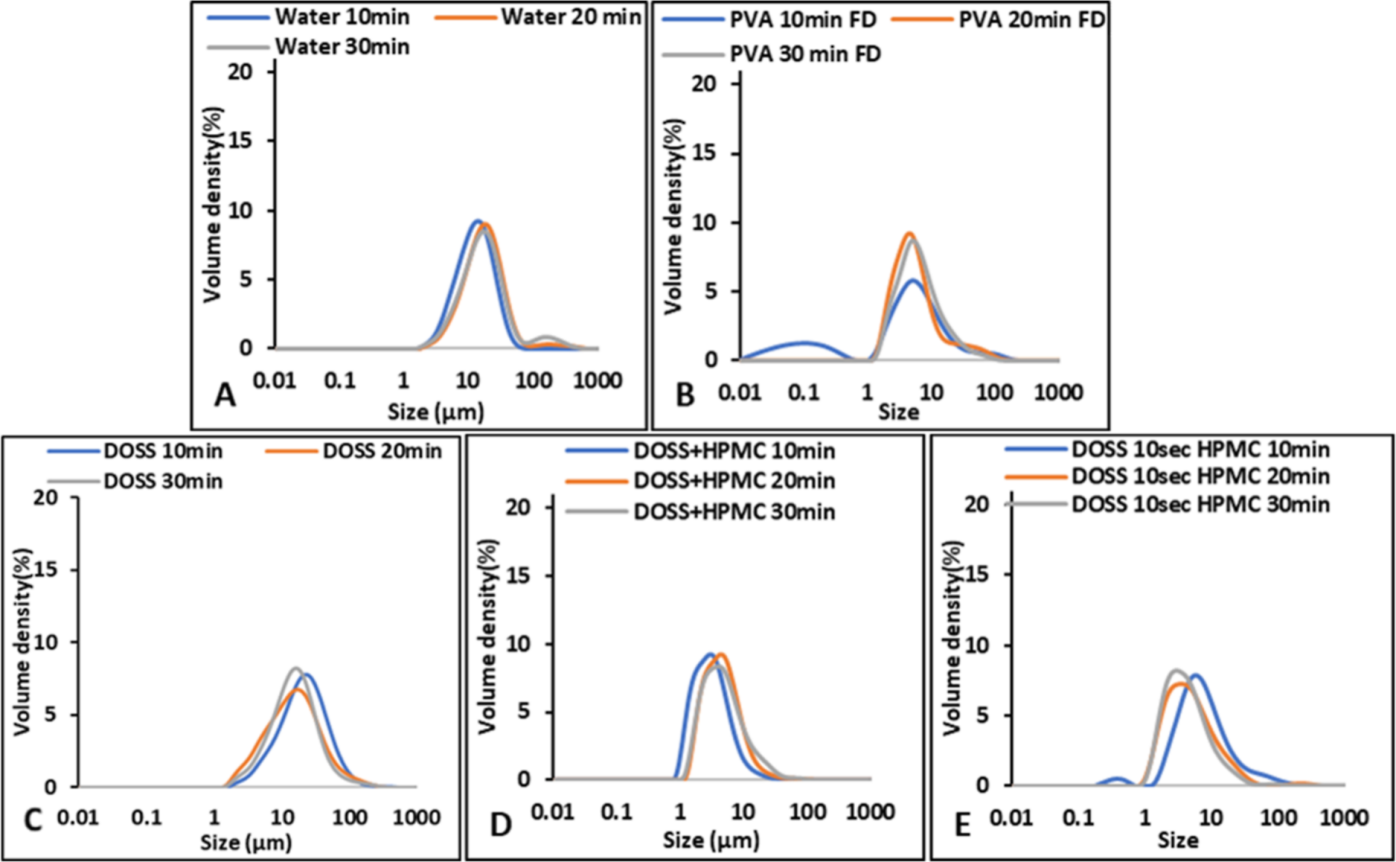
PSD of resuspended FD FF prepared at 5 °C and different aging times (A) without additives; (B,D) with single additive; and (D,E) with multiple additives.

**Figure 8:**
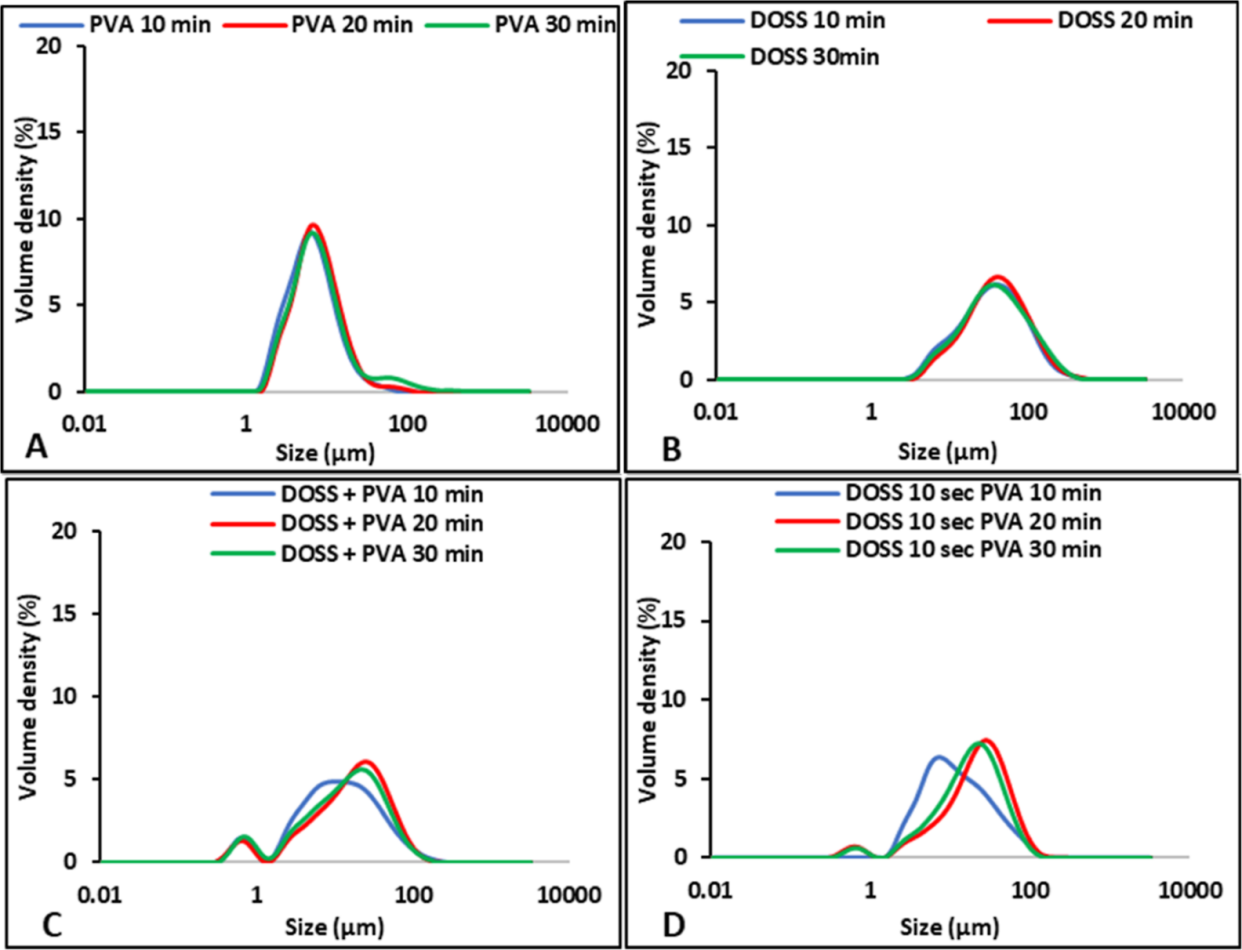
PSD of resuspended FD DCP prepared at 5 °C with different aging times (A,B) with single additive and (C,D) with multiple additives.

The effect of aging time on particle size observed in the fresh suspension, as shown in Figures 2 and 3, was indistinguishable in the resuspended FD API samples, if the AS crystallization process was followed by a freeze-drying process. This was observed for both FF and DCP with and without the additives, as shown in Figures 7 and 8.

Thus, from the PSD of both FD APIs, it can be concluded that freeze-drying promotes agglomeration, and the influence of aging time has disappeared.

### 3.3 Dissolution Behavior

**Figure 9:**
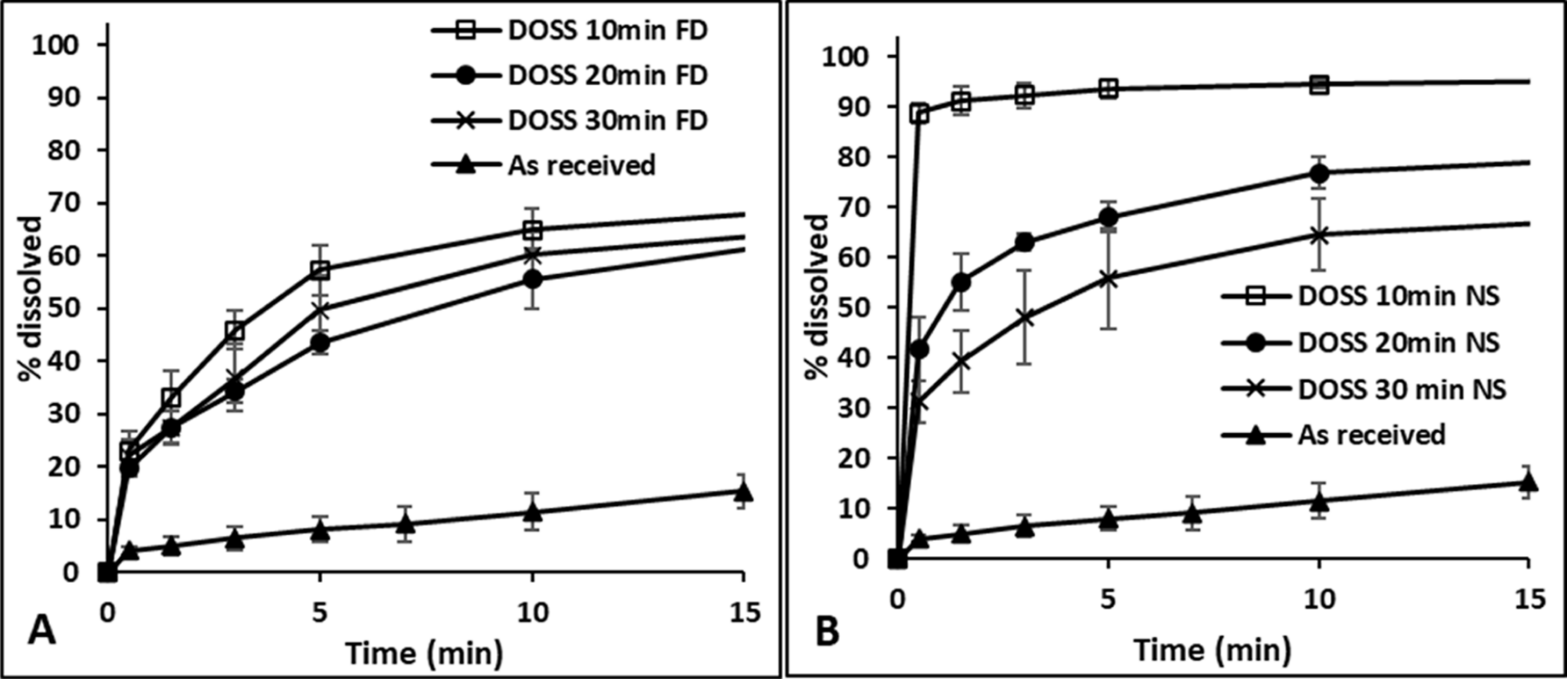
Dissolution profile of (A) FD FF powders and (B) freshly prepared FF suspensions precipitated in the presence of the single additive DOSS at 5 °C at 10, 20, and 30 min aging times, respectively. The dissolution profile for “as-received” FF is shown for reference. FD and NS correspond to FD and nanosuspension, respectively.

FF suspensions prepared with the single additive, DOSS, at 10, 20, and 30 min aging times showed distinct differences in their rates of dissolution, ranging from 93, 68, and 56% dissolution, respectively, within the initial 5 min, as shown in Figure 9B. These results can be correlated with their PSD showing an increase in particle size with increasing aging time. In contrast, the dissolution profiles of FD FF samples precipitated in the presence of DOSS at different aging times showed comparable dissolution profiles, that is, reaching ∼63% dissolution after 10 min, as shown in Figure 9A. No significant differences were observed in the extent of dissolution in FD FF samples prepared with 20 and 30 min aging times at all time points. The FD FF sample with 10 min aging time, however, did show a slightly faster extent of dissolution initially compared to the FD FF samples prepared with longer aging times, but the difference was much smaller compared to the impact of aging time on the dissolution of the freshly prepared suspensions, as shown in Figure 9. Similar trends were observed for FF with PVA alone and for DCP with PVA or DOSS alone (Supporting Information).

**Figure 10:**
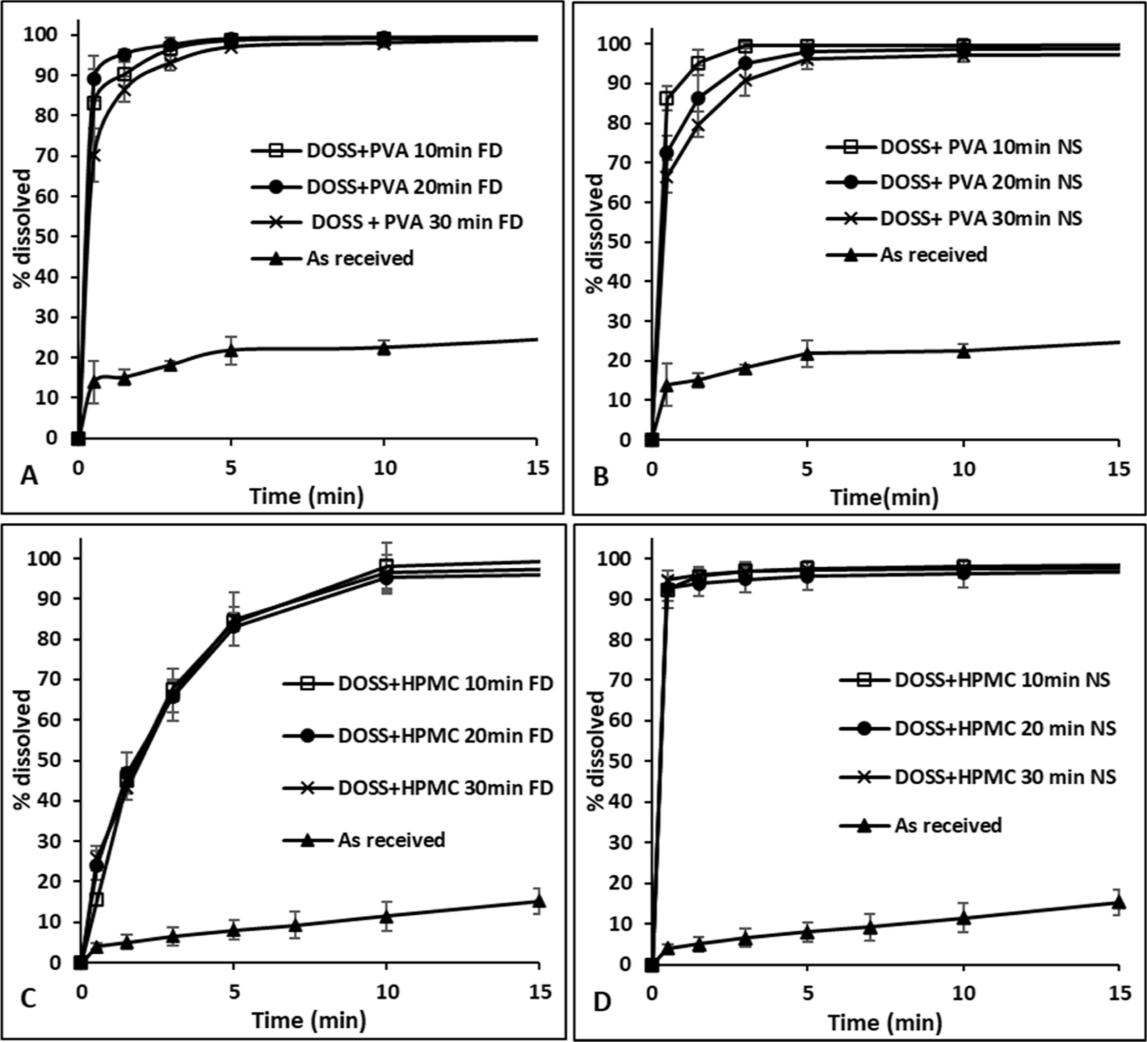
Dissolution profile of FD API powders and freshly prepared API suspensions in the presence of multiple additives at 10, 20, and 30 min aging times: (A,B) DCP and (C,D) FF. Dissolution profile for “as-received” APIs shown for reference. FD and NS correspond to FD and nanosuspension, respectively.

**Figure 11:**
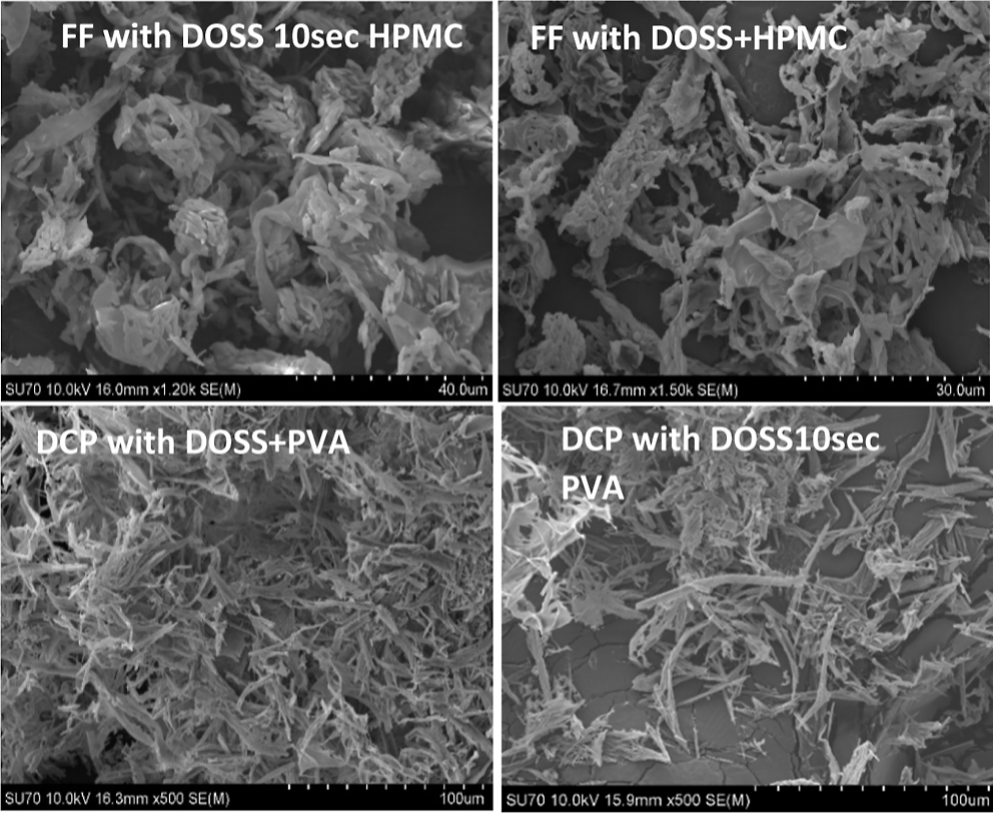
SEM images of FD API powders prepared in the presence of multiple additives.

For the API fresh suspensions generated in the presence of multiple additives, where particle growth was inhibited for 30 min, there was no impact on the dissolution profile with aging time, up to 15 min, as shown in Figure 10. Freeze-drying did reduce the rate of dissolution for FF over the first 10 min but not for DCP when multiple additives were used, as shown in Figure 10. FD or freshly prepared API suspensions precipitated with multiple additives at different aging times showed rapid dissolution rates with >95% of the API dissolving within 10 min, again with no significant differences being observed in the extent of dissolution at each time point for the different samples. Freshly prepared FF or DCP suspensions in the presence of DOSS + HPMC or DOSS + PVA, respectively, had stable nanometer-range particle sizes for up to 30 min aging time, as shown in Figure 5. Although the particle size of FD APIs precipitated in the presence of multiple additives was in the micrometer range (Figures 7 and 8), the particles had a thin flaky shape resulting in a large surface area (Figure 11 and Supporting Information) which contributed to high dissolution rates upon resuspension. Loose aggregation of the finer particles during drying, causing an increase in particle size to the micrometer range as measured by light-scattering analysis, may also have preserved the effective surface area and hence the dissolution was not adversely effected.

**Figure 12:**
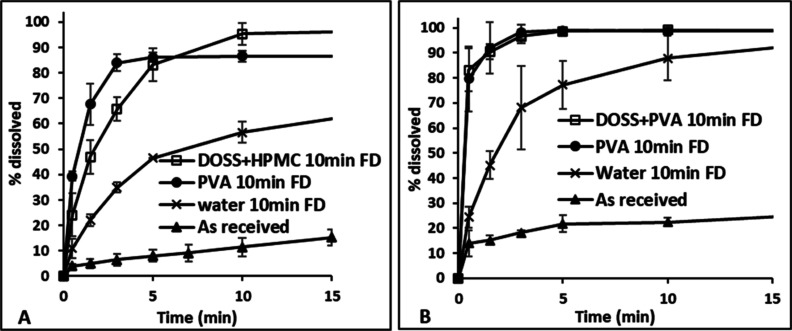
FD single and multiple additive systems that resulted in the fastest dissolution profiles in comparison with “as-received” and without additive systems: (A) FF and (B) DCP.

FD FF and DCP powders precipitated in the presence of PVA alone or the multiple additives DOSS + HPMC and DOSS + PVA, respectively, resulted in the fastest dissolution profiles, as shown in Figure 12. Although the dissolution rates were much higher in comparison to the “as-received” or FD without an additive system, there was not much difference between single or multiple additives. Thus, the advantage of generating stable suspensions in the presence of multiple additives compared to a single additive was lost during freeze-drying. All experiments reached 100% dissolution after 24 h.

## 4 Discussion

When undersaturated solutions of FF or DCP were added into an aqueous AS with rapid mixing, a high supersaturation level was obtained, resulting in high nucleation rates. Small, narrowly distributed particles were observed at 30 s aging time. For all experiments conducted, a more stable suspension in the nanometer range was obtained at 5 °C than at 25 °C which can be explained by the effect of temperature on nucleation rate and crystal growth. At lower temperatures, the solubility of the API is lower, resulting in higher supersaturation. There is competition between nucleation rate enhancement due to this increase in supersaturation and nucleation rate reduction due to slower kinetics at lower temperature. However, in the present work, the observed smaller particle size at a lower temperature indicates that the increase in supersaturation is the dominant influence. Lower temperature also helped to stabilize the suspensions over time, potentially due to a decrease in diffusion resulting in slower growth rates and less Ostwald ripening.^54,55^ Still, the lower temperature was still insufficient to stabilize the nanosuspensions, and agglomeration was observed at aging times longer than 20 min.

Among the AS crystallization experiments performed with single additives, PVA stabilized the particle sizes of both FF and DCP. PVA has numerous hydrogen-bond donor sites which can interact with the API hydrogen acceptor sites (FF molecules have four hydrogen-bond acceptor sites, while DCP has two) at the surface of the precipitated particles to stabilize the suspensions, as shown in Figure 1. Also, FF has only hydrogen-bond acceptors but no donors which contributed to the absence of API intermolecular or interparticle hydrogen bonds and can enhance the stabilizing effect of PVA. HPMC has both hydrogen-bond donor and acceptor sites but is reported to interact with FF by van der Waal interactions.^56,57^ The high viscosity of both HPMC and PVA solutions (Supporting Information) also helped to lower the diffusion rate, which possibly led to initial smaller particle sizes. With longer aging times, crystal growth occurs, overcoming the weak van der Waal interactions between HPMC and FF to form larger particles. At low temperature (5 °C), the enhancement of viscosity also contributes to a more stable FF nanosuspension for up to 10 min in the presence of HPMC or PVA.

DOSS is an anionic surfactant having two hydrophobic tails and a hydrophilic inner moiety, as shown in Figure 2. It can potentially adsorb to the surface of API nanoparticles via hydrophobic interactions and provide electrostatic stabilization.^21,58^ Changes in the zeta potential values of the particles with the different additives were observed from −34.9 mV (without additives) to −44 mV (with DOSS) and from −38.8 mV (without additives) to −58.5 mV (with DOSS) for FF and DCP, respectively. These changes in the surface charge of the suspended API particles confirm adsorption of the DOSS molecules to the surface of the particles. However, DOSS alone could not stabilize the nanosuspension over time. It is hypothesized that the polymeric additives, PVA and HPMC, are also adsorbed to the surface of the precipitated particles, which is supported by the reduction in zeta potential (Table 2) observed when they are included in the AS, resulting in steric stabilization of the particles in suspension.

**Table 2:** ζ Potential of API Nanosuspension Prepared in the Presence or Absence of Additives

API	system	ζ potential (mV)
FF	without additive	–35 ± 2
	DOSS	–44 ± 3
	PVA	–14 ± 1
	DOSS + HPMC	–4 ± 1
DCP	without additive	–39 ± 2
	DOSS	–59 ± 3
	PVA	–22 ± 2
	DOSS + PVA	–19 ± 2

The presence of both polymeric and surfactant additives showed a synergistic effect on the production and stabilization of FF suspensions.^57^ When comparing the results of PVA and DOSS with HPMC and DOSS, the latter combination resulted in a more stable nanosuspension, perhaps due to the fact that at low temperature, the viscosity (Supporting Information) of the suspension containing HPMC is larger than that with PVA, resulting in a more stable PSD. For stepwise addition, the surfactant additive DOSS was present during nucleation, after which the polymeric additive HPMC was added to stabilize the nanoparticles. For FF with these two additives, stepwise addition of additives did not provide any further stabilization or destabilization in PSD compared with the presence of both additives during nucleation.

DCP molecules have two hydrogen-bond acceptors and one hydrogen-bond donor site. It interacts similar to FF with additives DOSS and PVA to produce stable nanosuspension for up to 30 min. The reason for HPMC stabilizing particle sizes better for FF than DCP in the presence of DOSS is not clear, but it may be due to the competing interactions of HPMC and PVA with different functional groups on the two selected APIs. From the experiments of simultaneous and stepwise addition of DOSS and PVA, both showed similar PSD for DCP, similar to FF. However, while HPMC and DOSS resulted in larger particles of DCP than PVA and DOSS, it is worth noting that the stepwise addition of DOSS first followed by HPMC after the nucleation process resulted in a narrower PSD than when both HPMC and DOSS were present at the time of nucleation, Supporting Information. This finding is similar to that reported by Bodnar et al.^21^ Nanosuspensions of both FF and DCP in the presence of multiple additives remained stable for up to 30 min only, and with increasing time, grew to microsuspensions.

DSC patterns of FD FF showed that partial polymorphic transformation of FF occurred without additive or with a single additive, whereas only stable form I was produced in the presence of multiple additives. This could be due to a faster transformation or, potentially, the direct nucleation of the stable form in the presence of multiple additives. The presence of the metastable form IIa was not detectable via PXRD in the FF powders containing single additives due to the low intensity of the form IIa peaks generated from the small amounts present. Solid-state analysis, that is, PXRD and DSC, of all FD DCP particles showed the same active pharmaceutical form, form A.

Dissolution rates of dry samples depend upon the dispersibility, wettability, particle size, and the surface area of the API particles that comes directly in contact with the DM.^9,56^ “As-received” APIs showed the lowest dissolution rate due to their larger particle size and smaller surface area (Supporting Information). In the presence of additives, the particles having hydrophilic moieties from the additives bound to the surface of the API particles were observed to suspend easily in the aqueous DM indicating an increase in the wettability of the FD particles. The dissolution rate of both FD API particles precipitated in the presence of additives showed higher dissolution rates compared to FD particles precipitated without additives. This difference in dissolution rates can be attributed to poorer wettability and larger particle size in the absence of additives. FD FF powders with multiple additives had high dissolution rates similar to freshly prepared FF nanosuspensions. Although freeze-drying of FF nanosuspensions led to the formation of larger agglomerates, it was observed in this work during dissolution studies that the presence of additives enhanced the redispersibility of the dried particles resulting in high dissolution rates. Thus, the effect of aging time with single additives on dissolution was negligible for FF if followed by a freeze-drying process.

Dissolution studies of all FD DCP particles with additives with different particle sizes (Supporting Information) showed rapid dissolution rates. From the SEM images, FD DCP particles in the absence of additives have plate like habits similar to “as-received” DCP (Supporting Information), whereas in the presence of additives, most had thin flaky or needlelike habits (Supporting Information). This type of habit leads to higher specific surface areas and thus high dissolution rates. All FD DCP samples had high dissolution rates and reached ∼98–100% within the first 30 min, which was quite similar to the dissolution profile of the freshly prepared nanosuspensions, as shown in Figure 10A,B and Supporting Information.

## 5 Conclusions

The mechanism by which additives such as surfactants or polymers stabilize the PSD and prevent agglomeration of an API suspension during an AS crystallization is not clearly understood. In the cases of the APIs explored during this work, the presence of both surfactant- and polymeric-type additives during AS crystallization or having just the surfactant additive present during nucleation followed by addition of the polymeric additive showed no considerable difference in particle size with the exception of the combination of HPMC and DOSS with DCP. In addition to this, the ability of the additives that successfully stabilize an API suspension to continue to retain the API small particle sizes through subsequent processing steps, such as drying, is also unknown. This work showed that multiple additives were better than single additives at stabilizing small API particle sizes in suspension before the freeze-drying process. After freeze-drying, however, powders produced with single or multiple additives showed similar flaky particle habits and similar fast dissolution profiles, negating the advantage of the smaller particle sizes observed prior to the drying process with the multiple additives. The polymorphic purity of the dried powders, however, was higher in the presence of multiple additives than in the presence of single additives.

Ultimately, size reduction is a formulation approach taken to improve the dissolution rate and solubility of poorly water-soluble drugs. Thus, this work shows that the selection of additives to control particle size during crystallization should also be examined for their impact on wettability, polymorphic purity, and crystal habit, in addition to the particle size, during subsequent processing steps such as drying.

## Supporting Information Available

Additional PSD of API suspensions prepared in the presence of single and multiple additives, particle size values of all API suspensions prepared at different aging times, viscosity measurement of polymeric additives, DSC analysis of additives, additional SEM images of “as-received” and FD APIs, and additional dissolution profile data for freshly prepared and FD APIs (PDF)
