# Supplementary material for: Impact of Additives on Drug Particles during Liquid Antisolvent Crystallization and Subsequent Freeze-Drying
Source: Org Process Res Dev. 2023 Oct 6;27(11):2020–34. doi: 10.1021/acs.oprd.3c00204 (PMC10661054; doi:10.1021/acs.oprd.3c00204)
Supplement: Supplementary file 1 — op3c00204_si_001.pdf [file op3c00204_si_001.pdf]

Impact of additives on drug particles during liquid antisolvent crystallization and subsequent freeze-drying

Peuli Ghosh[a], Ake Rasmuson[a,b] and Sarah P. Hudson[a]*

[a]*SSPC, the Science Foundation Ireland Research Centre for Pharmaceuticals, Department of Chemical Sciences, Bernal Institute, University of Limerick, Limerick, V94 T9PX, Ireland*

[b]*Department of Chemical Engineering, KTH Royal Institute of Technology, Stockholm, SE-100 44, Sweden*

*Email: sarah.hudson@ul.ie

Supporting Information

Contents

1. Additional Particle Size Distributions of API Suspensions 2

1.1. Particle Size Distributions of API suspensions in presence of HPMC 2

1.2 Particle Size Distributions of API suspensions with multiple additives 2

1.3. Particle size values of API suspensions prepared without additive 3

1.4. Particle size values of API suspensions prepared in the presence of a single additive 4

1.5. Particle size values of API suspensions prepared in the presence of multiple additives 6

2. Viscosity measurement of polymeric additives 8

3. DSC Analysis of Additives 9

4. SEM and Particle Size Analysis of FD API Powders 9

4.1 SEM and Particle Size Analysis of 'as received' APIs 9

4.2. Particle size values of freeze dried dalcetrapib powder 10

4.3. SEM images of freeze dried APIs prepared without additive 10

4.5. SEM images of freeze dried APIs in the presence of multiple additives 11

5. Dissolution Profiles 12

5.1. Dissolution profile comparison of freeze dried and freshly prepared DCP in the presence of PVA 12

5.2. Dissolution profile comparison of freeze dried and freshly prepared DCP in the presence of a single additive 13

1. Additional Particle Size Distributions of API Suspensions

1.1. Particle Size Distributions of API suspensions in presence of HPMC

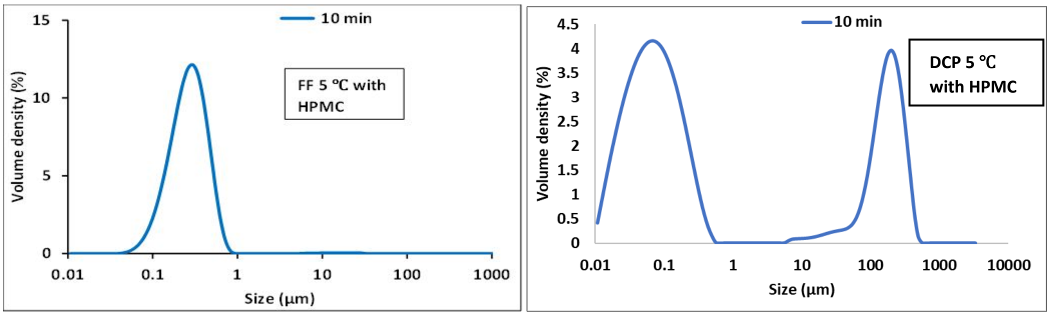

Figure S1. Particle size distribution of FF and DCP suspensions prepared with HPMC at 5˚C and 10 min aging time. The particles settled out of suspension at longer aging times.

1.2 Particle Size Distributions of API suspensions with multiple additives

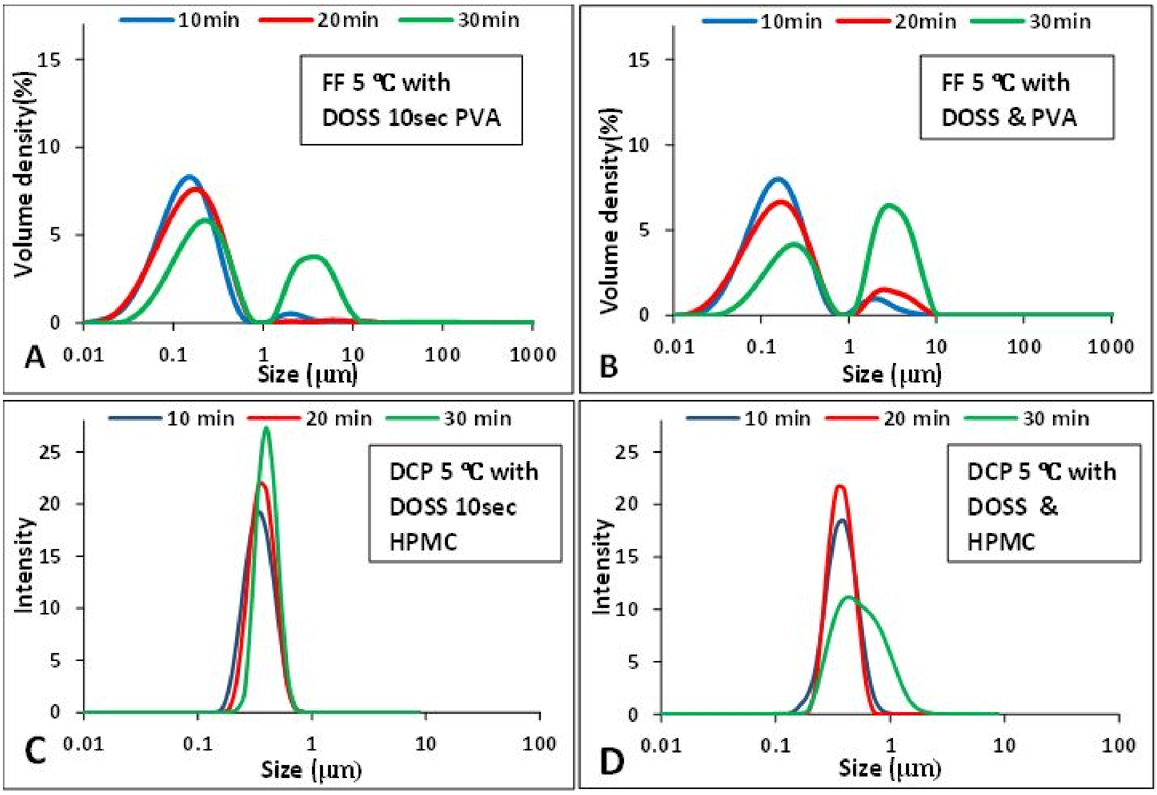

Figure S2. Particle size distribution of API suspensions at 5˚C over time (A) FF with DOSS 10sec PVA, (B) FF with DOSS & PVA, (C) DCP with DOSS 10sec HPMC, (D) DCP with DOSS & HPMC

1.3. Particle size values of API suspensions prepared without additive

Table S1. Particle size values over time for fenofibrate and dalcetrapib in absence of additives. (n= 3 for each sample measurement)

API	Temperature	Additive	Time	D[10] µm	D[50] µm	D[90] µm
Fenofibrate	20 ℃	Without additive	0.5 min	0.2±0.1	0.4±0.1	0.5±0.1
			10 min	0.6±0.4	23.0±3.4	79.0±5.6
			20 min	-------	-------	-------
			30 min	-------	-------	-------
	5 ℃	Without additives (AS/S – 10:1)	0.5 min	0.04±0.01	0.3±0.1	0.8±0.1
			10 min	0.6±0.1	4.3±3.8	43±5
			20 min	-------	-------	-------
			30 min	-------	-------	-------
		Without additives (AS/S – 47:3)	0.5 min	0.02±0.01	0.08±0.10	0.7±0.1
			10 min	0.04±0.01	0.2±0.1	30±1
			20 min	-------	-------	-------
			30 min	-------	-------	-------
Dalcetrapib	25 ℃	Without additives	0.5 min	0.1±0.1	0.3±0.1	0.7±0.1
			10 min	-------	-------	-------
			20 min	-------	-------	-------
			30 min	-------	-------	-------
	5 ℃	Without additives	0.5 min	0.03±0.1	0.1±0.1	0.2±0.1
			10 min	0.03±0.04	0.08±0.04	0.3±0.2
			20 min	0.03±0.02	0.2±0.1	55±5
			30 min	-------	-------	-------

1.4. Particle size values of API suspensions prepared in the presence of a single additive

Table S2. Particle size values over time for fenofibrate and dalcetrapib in the presence of a single additive. (n= 3 for each sample measurement)

API	Temperature	Additive	Time	D[10] µm	D[50] µm	D[90] µm
Fenofibrate	20 ℃	PVA	0.5 min	---------	0.34±0.01*	--------
			10 min	0.1±0.1	0.3±0.1	1.5±0.9
			20 min	0.8±0.1	3.2±0.9	5.3±1.6
			30min	1.9±0.9	3.7±1.1	5.8±2.2
		DOSS	0.5 min	-------------	0.27±0.01*	-----------
			10 min	0.2±0.1	0.9±0.1	3.6±0.4
			20 min	6.4±0.6	21.0±1.3	48.0±4.6
			30 min	16.0±2.8	39.0±3.4	71.0±3.7
		SDS	10 min	0.1±0.1	0.2±0.1	12.1±3.6
			20 min	9.6±0.1	28.1±2.4	55.4±4.3
			30 min	--------	--------	--------
		PVP K-30	10 min	0.2±0.1	0.3±0.1	0.6±0.1
			20 min	0.2±0.1	0.4±0.1	0.7±0.1
			30 min	0.2±0.1	7±5	206±36
	5 ℃	PVA	0.5 min	---------	0.27±0.02*	--------
			10min	0.2±0.1	0.3± 0.1	0.60±0.14
			20min	0.2±0.1	0.4±0.1	0.7±0.1
			30min	0.2±0.1	0.4±0.1	2.2±0.2
		DOSS	0.5 min	----------	0.20±0.01*	----------
			10 min	0.1±0.1	0.2±0.1	0.5±0.1
			20 min	0.2±0.1	0.3±0.1	2.1±1.5
			30 min	0.2±0.1	5.0±2.3	19.0±7.4
Dalcetrapib	25 ℃	PVA	0.5 min	-----------	0.36±0.03*	---------

			10 min	0.1±0.1	0.3±0.1	1.2±0.8
			20 min	0.2±0.1	0.4±0.1	1.0±0.6
			30 min	0.5±0.1	10±2	4±7
		DOSS	0.5 min	------------	0.29±0.02*	----------
			10 min	0.03±0.01	0.1±0.1	0.6±0.1
			20 min	0.1±0.1	2.4±0.8	22.7±4
			30 min	11±2	36±8	88±14
		PF127	10 min	0.1±0.1	0.2±0.1	0.5±0.1
		PVP K30	10 min	0.1±0.1	0.3±0.1	0.6±0.1
		Tween 80	10 min	0.1±0.1	0.2±0.1	0.5±0.1
	5 ℃	PVA	0.5 min	-------------	0.23±0.02*	--------
			10 min	0.02±0.01	0.08±0.01	0.3±0.1
			20 min	0.02±0.01	0.08±0.01	0.3±0.1
			30 min	0.02±0.01	0.1±0.1	3±1
		DOSS	0.5 min	-------------	0.19±0.01*	------------
			10 min	0.02±0.01	0.07±0.01	0.2±0.1
			20 min	0.02±0.01	0.09±0.04	22±8
			30 min	0.03±0.01	0.2±0.1	56±11

*Particle size for this nanosuspensions were measured by Zetasizer

1.5. Particle size values of API suspensions prepared in the presence of multiple additives

Table S3. Particle size values over time for fenofibrate and dalcetrapib in the presence of multiple additives. (n= 3 for each sample measurement)

API	Temperature	Additive	Time	D[10] µm	D[50] µm	D[90] µm
Fenofibrate	20 ℃	PVA & DOSS	0.5 min	-----------	0.26±0.02*	----------
			10 min	0.1±0.1	0.3±0.1	0.5±0.9
			20 min	0.1±0.1	1.4±0.6	6.0±1.8
			30 min	2.0±0.6	4.0±1.2	7.0±2.4
		DOSS 10sec PVA	0.5 min	------------	0.27±0.02*	-----------
			10 min	0.08±0.01	0.20±0.05	0.40±0.07
			20 min	0.1±0.1	0.4±0.2	5.0±1.8
			30 min	3.1±1.1	4.7±1.6	6.8±1.8
	5 ℃	PVA & DOSS	0.5 min	------------	0.21±0.02*	---------
			10 min	0.05±0.01	0.2±0.1	0.5±0.1
			20 min	0.05±0.01	0.2±0.1	2.3±1.2
			30 min	0.1±0.3	1.5±0.9	5±1.7
		DOSS 10sec PVA	0.5 min	-----------	0.20±0.02*	------------
			10 min	0.05±0.01	0.1±0.1	0.4±0.1
			20 min	0.08±0.01	0.2±0.1	0.4±0.1
			30 min	0.1±0.1	0.3±0.1	4.8±0.9
		HPMC & DOSS	0.5 min	------------	0.31±0.02*	----------
			10 min	0.1±0.1	0.2±0.1	0.3±0.1
			20 min	0.2±0.1	0.2±0.1	0.4±0.1
			30 min	0.2±0.1	0.3±0.1	0.4±0.1
		DOSS 10sec	0.5 min	-----------	0.29±0.02*	------------

		HPMC	10 min	0.1±0.1	0.2±0.1	0.3±0.1
			20 min	0.1±0.1	0.2±0.1	0.4±0.1
			30 min	0.1±0.1	0.3±0.1	0.5±0.1
Dalcetrapib	25 ℃	PVA & DOSS	0.5 min	-----------	0.27±0.02*	------------
			10 min	0.03±0.01	0.1±0.1	0.5±0.1
			20 min	0.2±0.1	0.9±0.1	20±6
			30 min	0.8±0.1	6.0±1.3	24±4
		DOSS 10sec PVA	0.5 min	------------	0.27±0.01*	------------
			10 min	0.03±0.1	0.1±0.1	0.4±0.1
			20 min	0.2±0.1	0.5±0.3	15±3
			30 min	0.6±0.1	8±2	29±7
	5 ℃	DOSS 10sec PVA	0.5 min	-------	0.21±0.02*	-------
			10 min	------	0.23±0.01*	-----
			20 min	-------	0.25±0.01*	-------
			30 min	--------	0.25±0.01*	-------
			60 min	0.9±0.1	5.5±2.9	14.8±3.4
		DOSS & PVA	0.5 min	-------	0.19±0.02*	-------
			10 min	-------	0.23±0.01*	------
			20 min	-------	0.24±0.01*	------
			30 min	--------	0.28±0.01*	-------
			60 min	0.8±0.1	4.8±2.6	14.1±3.2
		DOSS 1min PVA	10 min	--------	0.21±0.01*	---------
			20 min	--------	0.23±0.01*	--------

			30 min	0.02±0.01	0.08±0.01	0.3±0.08
		DOSS 5min PVA	10 min	--------	0.20±0.03*	--------
			20 min	--------	0.22±0.02*	--------
			30 min	0.03±0.01	0.18±0.10	7.2±1.3
		DOSS 10sec HPMC	0.5 min	------	0.28±0.03*	------
			10 min	------	0.34±0.02*	------
			20 min	------	0.35±0.02*	------
			30 min	------	0.39±0.02*	------
		DOSS & HPMC	0.5 min	------	0.31±0.02*	------
			10 min	------	0.35±0.03*	------
			20 min	------	0.37±0.02*	------
			30 min	------	0.42±0.05*	------

*Particle size for this nanosuspensions were measured by Zetasizer

2. Viscosity measurement of polymeric additives

The viscosity of prepared AS with single polymeric additives was measured by Brookfield Ametek rheometer with SC4 -13RP spindle at different temperature i.e, 5℃ and 25℃. The torque for each measurement was above 10%.

Table S4. Viscosity of polymeric additive solutions at 5 and 25 ℃

Temperature (℃)	Viscosity (cP)	
	PVA (1mg/ml)	HPMC (1 mg/ml)
5	1.75±0.02	1.99±0.03
25	1.26±0.05	1.38±0.02

3. DSC Analysis of Additives

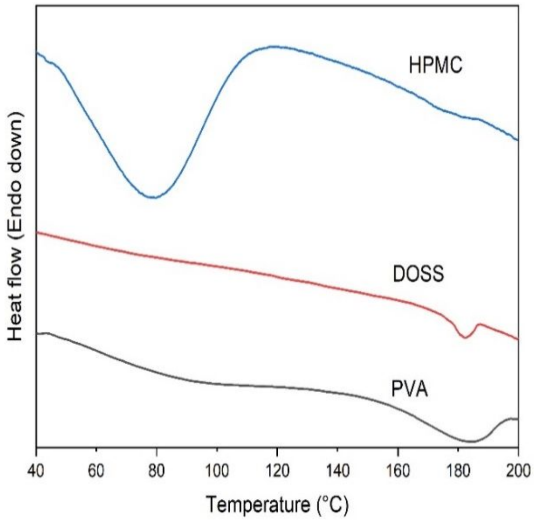

Figure S3. DSC analysis of as received additives.

4. SEM and Particle Size Analysis of FD API Powders

4.1 SEM and Particle Size Analysis of 'as received' APIs

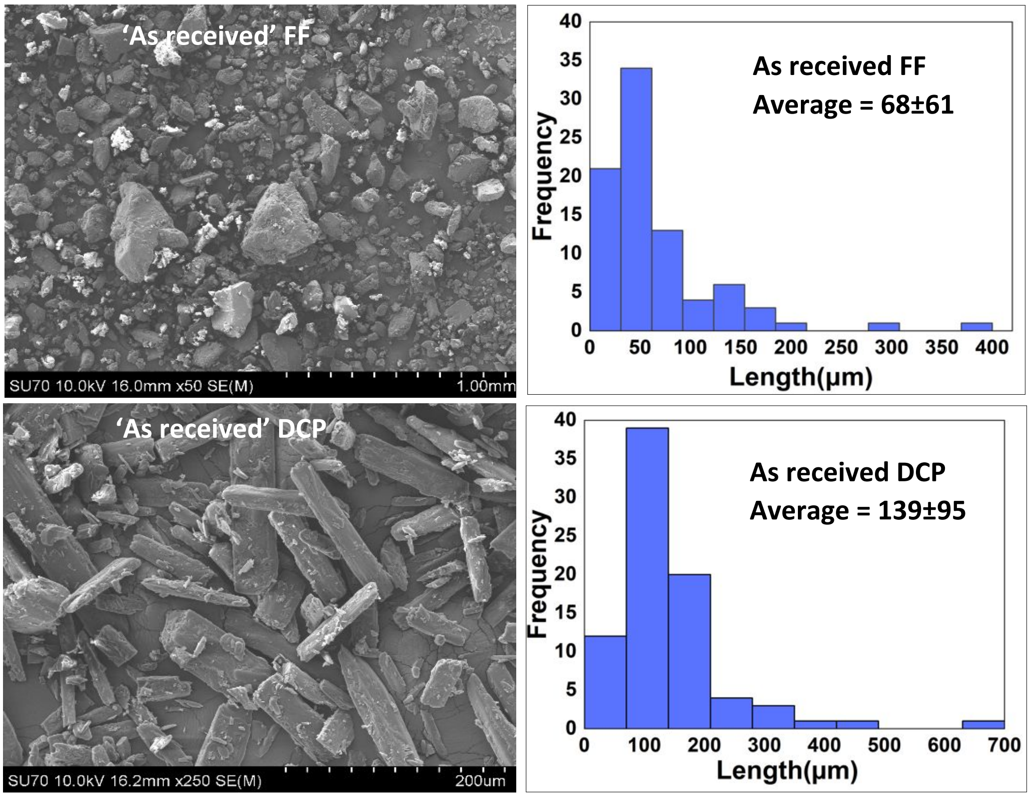

Figure S4. SEM images and particle size distributions of 'as received' FF and DCP powders measured from the SEM images using ImageJ.

4.2. Particle size values of freeze dried dalcetrapib powder

Table S5. Particle size values for freeze dried dalcetrapib in the presence of additives measured with the Mastersizer after resuspension in water (n= 3).

Additive system	D[50] (µm)<br>At 10 min aging time	D[50] (µm)<br>At 20 min aging time	D[50] (µm)<br>At 30 min aging time
PVA	5.0±2.2	7.0±3.5	7.0±2.3
DOSS	35.0±6.5	40.0±8.6	38.0±7.6
DOSS+PVA	11.0±3.7	18.0±4.4	15.0±4.5
DOSS 10sec PVA	11.0±2.8	13.0±3.8	19.0±7.7

4.3. SEM images of freeze dried APIs prepared without additive

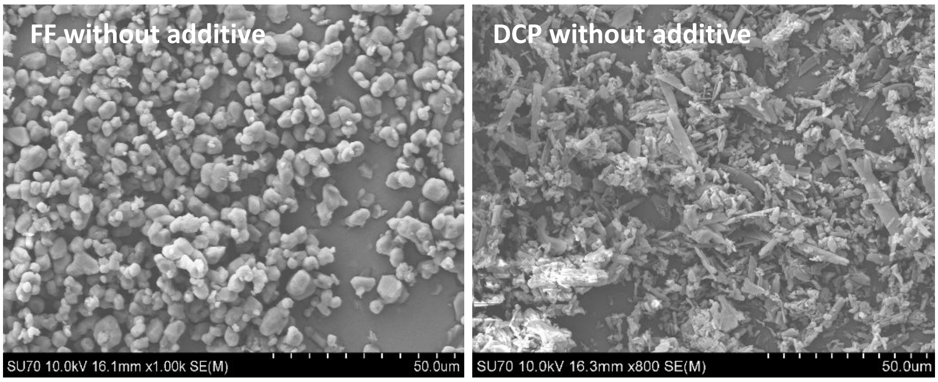

Figure S5. SEM images of freeze-dried APIs precipitated in absence of additive at 5 °C.

4.4 SEM images of freeze dried DCP in the presence of a single additive

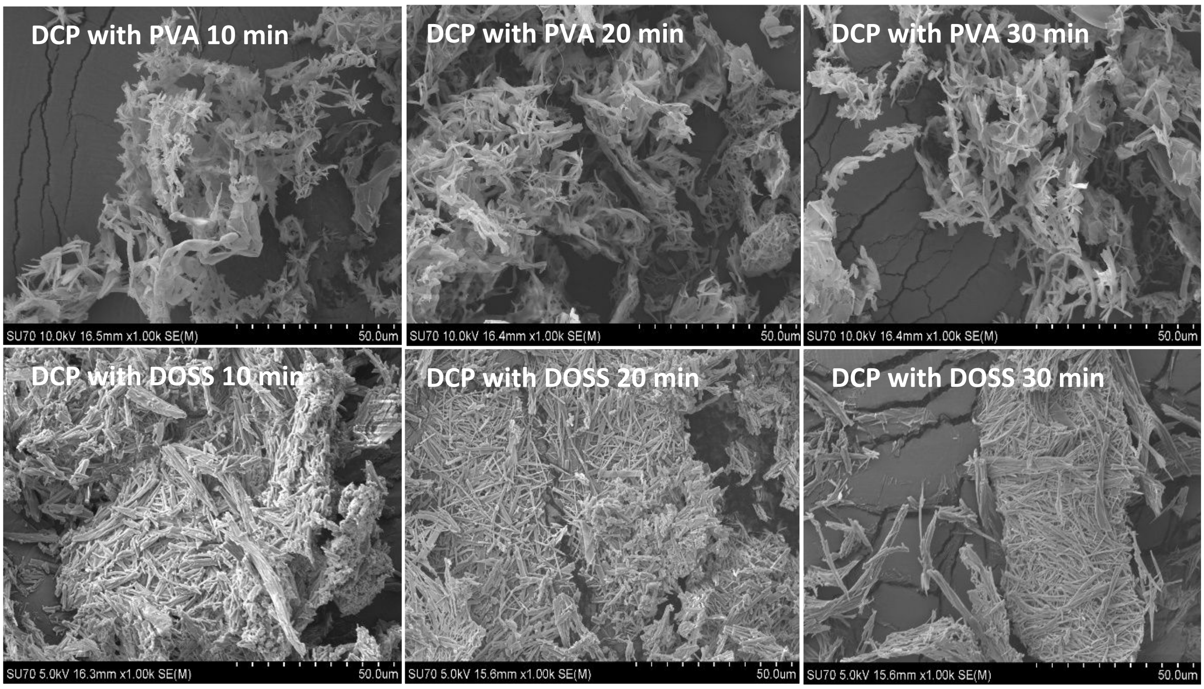

Figure S6. SEM images of freeze-dried DCP precipitated in the presence of a single additive at different aging time - 10 min, 20 min, 30 min at 5 °C.

4.5. SEM images of freeze dried APIs in the presence of multiple additives

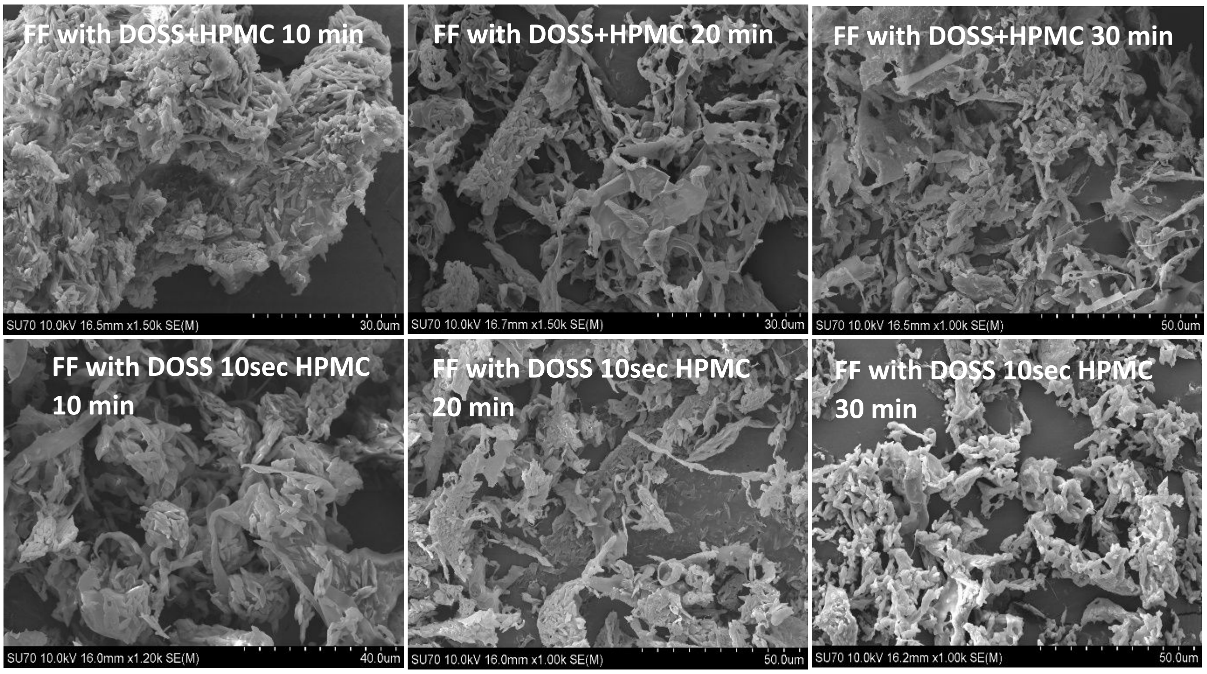

Figure S7. SEM images of freeze-dried FF precipitated in the presence of multiple additives at different aging time - 10 min, 20 min, 30 min at 5 ℃

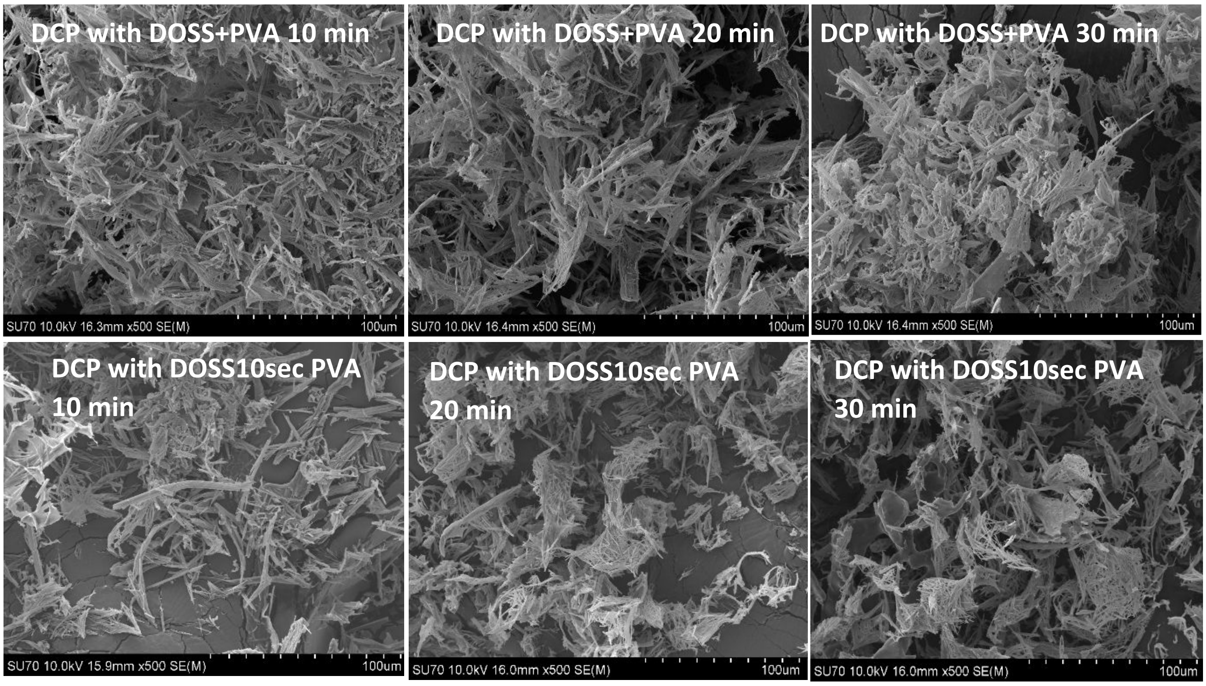

Figure S8. SEM images of freeze-dried DCP precipitated in the presence of multiple additives at different aging time - 10 min, 20 min, 30 min at 5 ℃

5. Dissolution Profiles

5.1. Dissolution profile comparison of freeze dried and freshly prepared DCP in the presence of PVA

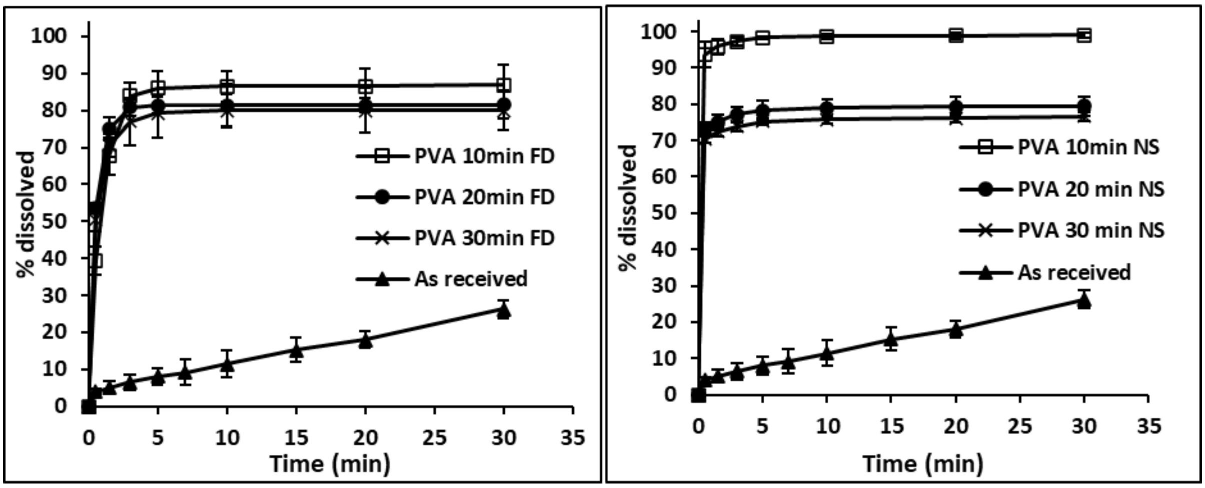

Figure S9. Dissolution profile of freshly prepared or freeze-dried FF precipitated in the presence of the single additive PVA at 5 °C for 10, 20, 30 min aging time respectively. The dissolution profile for 'as received' FF is shown for reference.

5.2. Dissolution profile comparison of freeze dried and freshly prepared DCP in the presence of a single additive

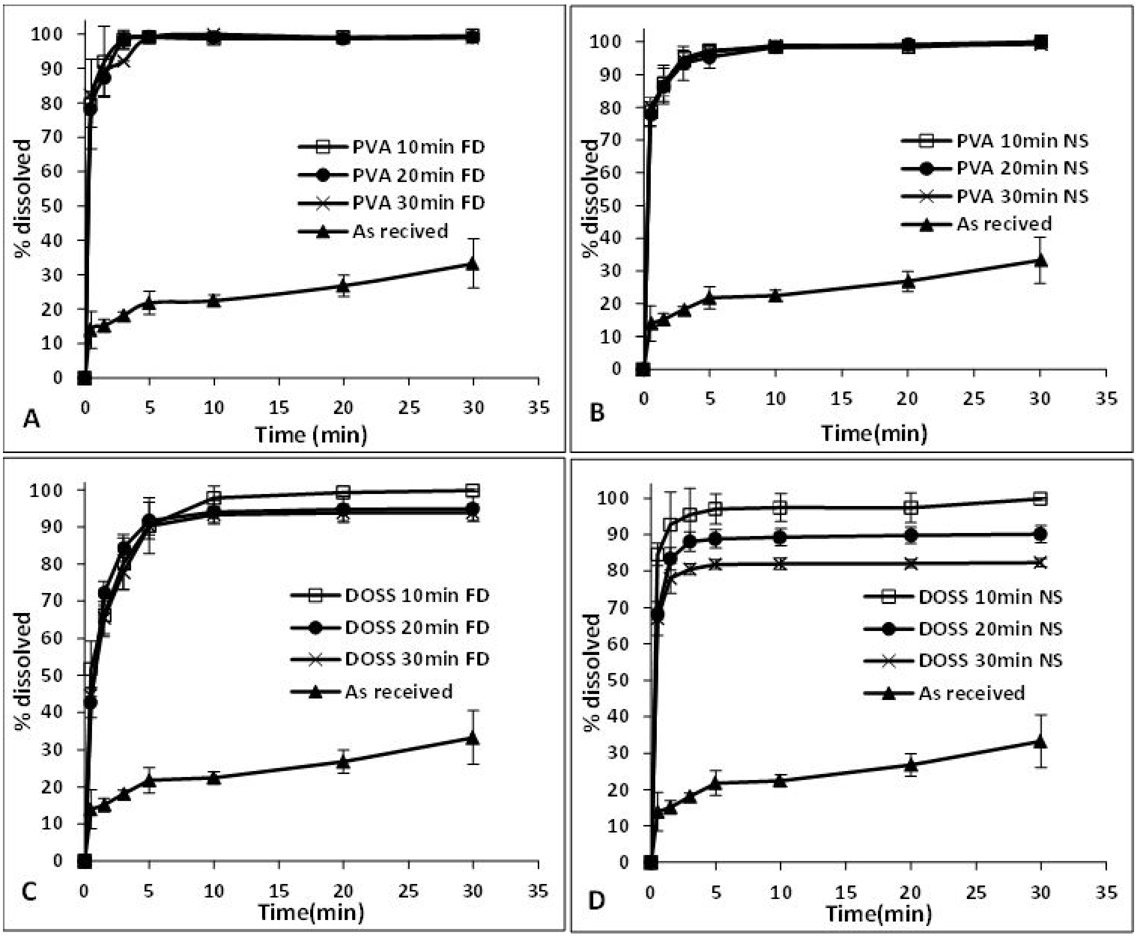

Figure S10. Dissolution profiles of (a, c) freeze-dried or (b, d) freshly prepared DCP nanoparticles prepared in the presence of a single additive, PVA or DOSS, at 10, 20, 30 min aging time respectively. Dissolution profile for ‘as received’ DCP shown for reference.
